# Tissue-resident stem cell activity: a view from the adult *Drosophila* gastrointestinal tract

**DOI:** 10.1186/s12964-017-0184-z

**Published:** 2017-09-18

**Authors:** Qiang Liu, Li Hua Jin

**Affiliations:** Department of Genetics, College of Life Sciences, Northeast Forestry University, No.26 Hexing Road Xiangfang District, Harbin, 150040 China

**Keywords:** *Drosophila*, Gastrointestinal tract, Stem cells, Proliferation and differentiation, Homeostasis

## Abstract

The gastrointestinal tract serves as a fast-renewing model for unraveling the multifaceted molecular mechanisms underlying remarkably rapid cell renewal, which is exclusively fueled by a small number of long-lived stem cells and their progeny. Stem cell activity is the best-characterized aspect of mucosal homeostasis in mitotically active tissues, and the dysregulation of regenerative capacity is a hallmark of epithelial immune defects. This dysregulation is frequently associated with pathologies ranging from chronic enteritis to malignancies in humans. Application of the adult *Drosophila* gastrointestinal tract model in current and future studies to analyze the immuno-physiological aspects of epithelial defense strategies, including stem cell behavior and re-epithelialization, will be necessary to improve our general understanding of stem cell participation in epithelial turnover. In this review, which describes exciting observations obtained from the adult *Drosophila* gastrointestinal tract, we summarize a remarkable series of recent findings in the literature to decipher the molecular mechanisms through which stem cells respond to nonsterile environments.

## Background

*Drosophila* is an excellent model system, due in large part to the ease of its genetic manipulation, that allows researchers to investigate prolonged intestinal inflammation and damage. The proliferative activity of a dedicated population of intestinal stem cells (ISCs) is instigated by a multitude of stresses and ensures the control of remarkably rapid cell renewal [1, 2]. Thus, to function efficiently, the adult *Drosophila* gastrointestinal tract possesses tools to maintain homeostasis and organismal health [3–6]. As recently established by a growing body of literature, these tools comprise a range of critical intestinal defense strategies, the dysregulation of which provokes the breakdown of intestinal homeostasis and precipitates or aggravates gastrointestinal diseases. (1) The intestinal lumen is lined by the peritrophic membrane, which represents the first line of host defense against invasion by enteric pathogens [7, 8]. (2) Rapid reactive oxide species (ROS) bursts, which are directly microbicidal, are triggered in epithelial cells following the ingestion of pathogens [9]. (3) In epithelial cells, Relish/NF-κB-dependent antimicrobial peptides (AMPs) are believed to act as a second line of defense for killing pathogens [10–14]. (4) The epithelial lining is rapidly regenerated in response to pathogens to maintain homeostasis [15]. ISCs that undergo mitosis give rise to differentiated cells and are responsible for a range of critical intestinal functions [16, 17].

Over decades of intensive study, research investigating the cues governing epithelial regenerative homeostasis has progressed. The ultimate goal of our review is to position recent discoveries within the context of how stem cells in the adult *Drosophila* gastrointestinal tract respond to environmental challenges.

## Review

### The adult *Drosophila* gastrointestinal tract: A comprehensive overview

#### Sequential organization

**Fig. 1:**
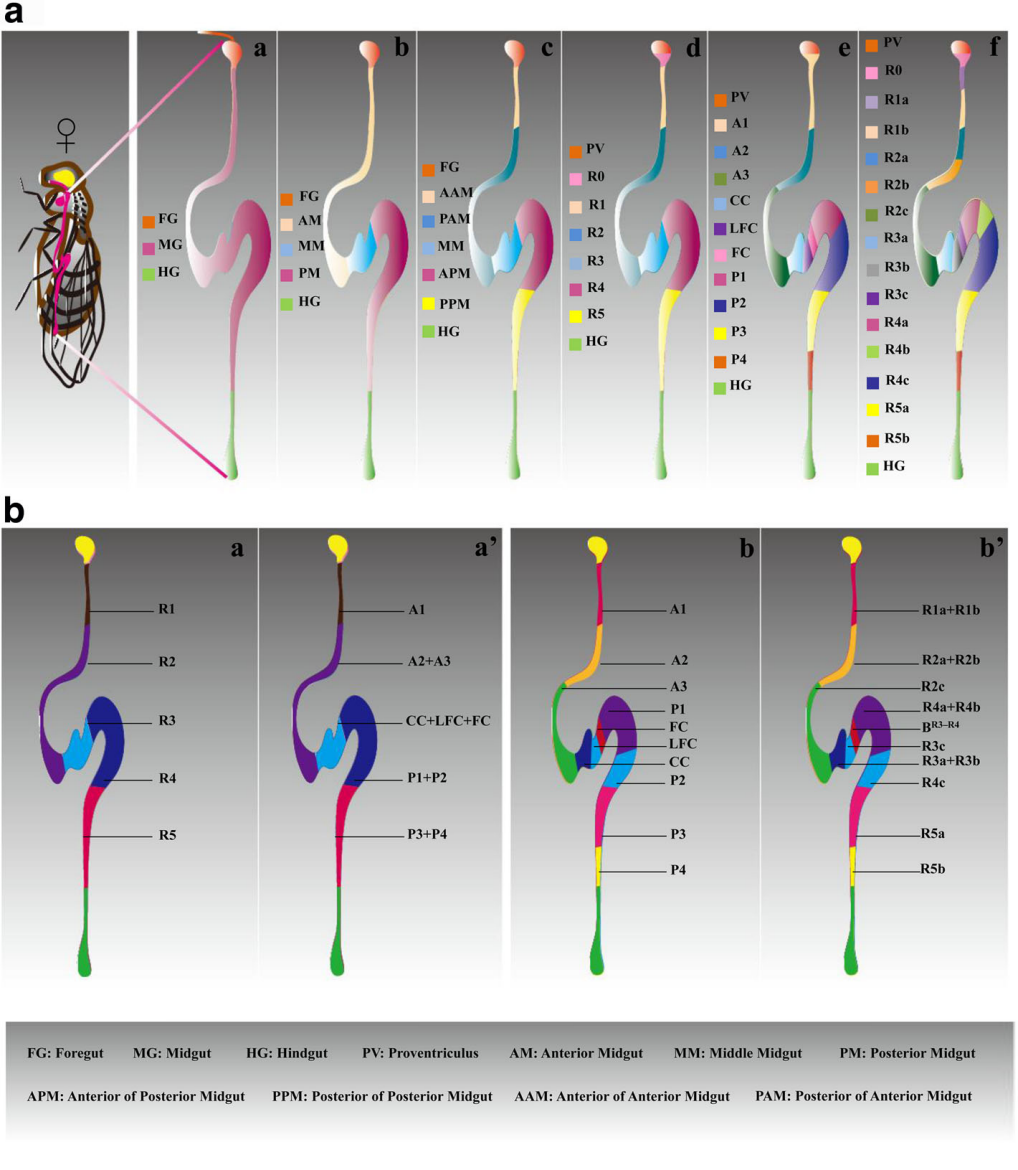
Atlases of sequential compartments. (**Aa**) Three discrete domains are defined: the FG, the MG and the HG. (**Ab**) The MG is divided into the AM, the MM and the PM. (**Ac**) The AM comprises the AAM and PAM; the PM comprises the APM and PPM. (**Ad, Ae**) Subdivisions (R0-R5 and A1-P4) are established. (**Af**) Thirteen subregions ranging from R1a to R5b represent the fine-grained compartmentalization of R0-R5. (**B**) The close correspondence between R0-R5 and A1-P4. B^R3-R4^ indicates the boundary of R3-R4. For example, R2 comprises A2 and A3 (**Ba, Ba′**), and A2 comprises R2a and R2b (**Bb, Bb’**)

First, this review will introduce the adult gut architecture. The anatomical details of the adult *Drosophila* gastrointestinal tract are relatively well known. It comprises a tubular epithelium consisting of three discrete domains with different developmental origins, cell types and physiological functions: the foregut, the midgut and the hindgut (Fig. 1Aa) [18–20]. (1) The foregut, which is lined by the impermeable cuticle, is derived from the embryonic ectoderm and is responsible for the transport and storage of ingested food [16, 21]. (2) The midgut, which absorbs nutrients, is of endodermal origin and is subdivided into three domains based on longitudinal pH gradients (Fig. 1Ab) [22]: the neutral segment, termed the anterior midgut (AM); the short and narrow middle midgut (MM) segment, which contains the copper cell region (CCR); and the wider, alkaline posterior midgut (PM), which has been the focus of a series of functional studies due to its physiological equivalence to the human small intestine. Further divisions of the AM and the PM are shown in Fig. 1Ac. (3) Reabsorption of water and the elimination of undigested waste are the responsibilities of the embryonic ectoderm-derived hindgut [21], which contains the pylorus, ileum and rectum. Additionally, the osmoregulatory and excretory apparatuses are the hindgut primordium and visceral mesoderm-derived Malpighian tubules (MTs), from which waste is released from the surrounding hemolymph into the gut lumen [23–26]. The MTs consist of the ureter, lower tubule and upper tubule [24].

The long-term maintenance of the integrity of the intestinal subregions is strongly associated with specialized physiological roles, the abnormal adjustment of which is characterized by a widespread loss of intestinal homeostasis. Thus, we next discuss current knowledge of the regionalization of the adult *Drosophila* intestine. Recently, the adult *Drosophila* gastrointestinal tract was shown to function as a workhorse for maintaining compartmentalization and region-specific regenerative activity. However, knowledge of the underlying gut regionalization is rudimentary. Conclusive evidence regarding the identity of subdivisions was recently described in two reports that independently arrived at corresponding conclusions and represented extraordinary achievements [27, 28] (Fig. 1b). Six distinct compartments designated region0-region5 (R0-R5) were identified by Lemaitre B and colleagues (Fig. 1Ad) [27]. Lemaitre B and colleagues also presented experimental evidence to accurately define the fine-grained regional organization of R1a-R5b (Fig. 1Af). Furthermore, loss of compartmentalization was closely coupled with the deregulation of homeostasis. This work demonstrated a correlation between gene expression domains and anatomical regions. For example, several transcription factors, including *caudal*, *pangolin*, *labial*, *Ptx1* and *GATA*, contribute to gut compartmentalization and ISC activity [27]. The transcriptomes of cells in each subregion were characterized by Buchon N, Edgar BA and colleagues, revealing the transcriptional diversity across R1-R5 [18]. Additionally, the impact of the microbiota on gene expression across R1-R5 has been described by Lemaitre B and colleagues [29]. For example, more microbiota-induced genes are up-regulated in R1 and R5 [29]. The same study also revealed the proportion of microbiota-induced genes in each gene ontology category per region [29]. A separate report defined ten distinct subregions of anterior1-posterior4 (A1-P4) in the context of regional differences in ISC properties [28] (Fig. 1Ae). Close correspondence between the two reports was confirmed by counting cell numbers based on the fractional length coordinates described by Lemaitre B and colleagues [27]. For example, A1 corresponded to R1a and R1b (Fig. 1Bb, b’), P1 corresponded to R4a and R4b (Fig. 1Bb, b’), R2 corresponded to A2 and A3 (Fig. 1Ba, a’) and R4 corresponded to P1 and P2 (Fig. 1Ba, a’). Altogether, these findings suggest that how regional boundaries are upheld during intestinal homeostasis is a question of great complexity. Importantly, the mammalian gut is also highly regionalized, both in terms of anatomy and function. Patterning of the intestinal crypts is established during development, and subregions are continually revised and maintained over a lifetime [30]. Therefore, the *Drosophila* gut serves as an excellent model to unravel the exact mechanisms responsible for such regionalization, providing a better understanding of gastrointestinal function in mammals.

To summarize, only a few studies have investigated gut subspecialization to date, and much of the fine-tuned regulation that controls compartmentalization is only beginning to be understood. Data obtained from these studies have opened other doors that lead to a wide spectrum of unsolved puzzles. For example, it is important to understand why the ISCs of P1 and A2 contain lipid droplets (shown below) [28], and it is not yet clear whether the lipid droplets within these ISCs play a special role in stem cell activity. Interestingly, with the exception of the large flat cell (LFC) region (LFCR) and Fe cell region (FCR), ISCs do not cross regional boundaries after division (termed “non-crossing” behavior) [28], but it is not clear how this occurs. Additionally, given that the sequential processing of ingested foods is regulated by different levels of digestive enzymes along the intestinal tube [27], we sought to explore the functional segmentation of intestinal cells among different subregions. One telling observation is that enterocyte (EC) morphology differs regionally, and R2-resident ECs contain lipid vesicles [28], prompting us to address the important question of whether ECs from different subregions possess the capacity to regulate different biological processes, such as metabolic homeostasis and stress responses. Finally, ISCs within P2 and P3 divide more rapidly than other regions during homeostasis [28]. Therefore, studies investigating ISC self-renewal have primarily focused on these regions. Given the different levels of Delta protein in ISCs across subregions described by another groundbreaking study [18], studies investigating differences in ISC activity across different subregions will also be of significant interest in the coming years.

#### Region- and organ-specific stem cells

As a starting point, the primary concern of studies investigating tissue regeneration and homeostasis is to validate the existence of enduring stem cells in rapidly self-renewing organs. This situation is progressively evolving, aided by two fascinating and pioneering studies demonstrating the maintenance of ISC-mediated homeostasis [1, 2]. The ISCs that reside in the AM and the PM are the primary stem cell type; these ISCs are continually exploited to investigate self-renewing and multipotent stem cell functions. According to Spradling AC and Marianes A, ISC morphology and the frequency of ISC division differ regionally [28]. Additionally, ISCs within P1 and A2 contain lipid droplets [28].

**Fig. 2:**
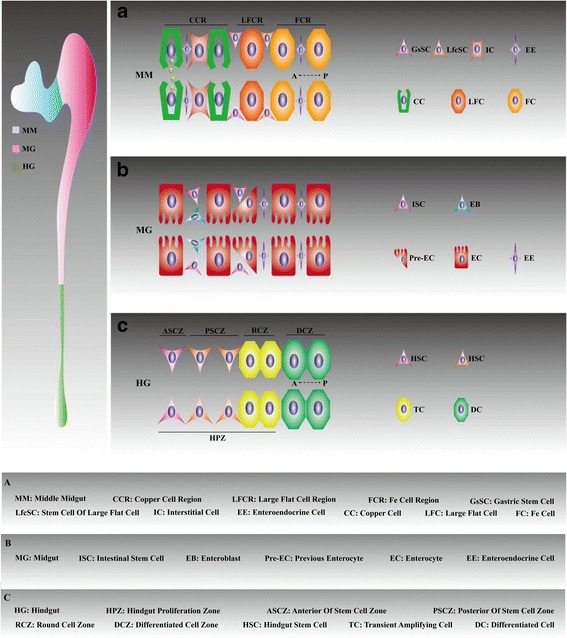
Epithelial architecture. **a** The lineages produced by GsSCs are composed of CCs, ICs and EEs. The LFCs and FCs are present in the posterior CCR. A: anterior, P: posterior. **b** The MG consists of two types of mature cells, with ISCs that are evenly distributed. **c** The HPZ is where damage-induced self-renewal is maintained by HSCs. Slow-cycling mode (ASCZ and PSCZ) is maintained by the HSCs in ASCZ. The TA cells in RCZ are involved in the differentiation of DCZ

Freshly armed with the discovery of ISCs, recent studies have sought to better characterize several other types of stem cells [31]. In this section, we review emerging data supporting the existence of five other stem cell types along the adult *Drosophila* gastrointestinal tract. (1) The discovery of hindgut stem cells (HSCs) was reported by two studies that obtained opposing results regarding HSC status. Hartenstein V and colleagues confirmed the existence of active stem cells [32]. In contrast, Spradling AC and Fox DT observed active HSCs within the hindgut proliferation zone (HPZ) in response to stress (Fig. 2c). However, HSCs are considered relatively quiescent in the absence of microbial pathogens [33]. (2) In the proventriculus (PV) region, gastric stem cells (GaSCs) are another type of stem cell [34]. Similar to HSCs, differentiated cells are derived from rapidly proliferating transient amplifying cells (TA cells) produced by GaSC division. (3) Based on evidence from Micchelli CA and Strand M, quiescent gastric stem cells (GsSCs) lie in the CCR region and regulate pathogen-induced regeneration [35]. However, GsSC status under homeostatic conditions is extremely controversial. Emerging data from a contradictory study suggest that GsSCs divide regularly every four to five days [28]. (4) The LFCR is also maintained by a small number of LFC stem cells (LfcSCs) [35] (Fig. 2a). (5) Additionally, a relatively fixed number of renal stem cells (RNSCs) in the lower MTs were identified by Hou SX and colleagues [36].

To summarize, these studies identify the following six main stem cell types: ISCs, HSCs, GaSCs, GsSCs, LfcSCs and RNSCs. Although HSCs and GsSCs are capable of undergoing mitosis, they do not appear sufficiently potent to be activated under homeostatic conditions. However, stress-induced damage triggers strong responses by these regional stem cells. LfcSCs and HSCs are marked by Delta and signal transducer and activator of transcription (STAT), respectively. The other stem cell types carry the unique general marker escargot (esg). A summary of these findings is provided in Table 1. This review next summarizes stem cell-mediated hierarchical organization.

**Table 1:** Summary of information for stem cell types

Types	Localizations	Status		Markers	Signaling		Discoverers	Year
		Conventional	Pathological		Proliferation	Differentiation		
GaSCs	PV	Active	Active	Wg, JAK-STAT, esg	JAK-STAT, Wg	Hh	Steven X. Hou	2011
GsSCs	CCR	Silent	Active	Delta, esg	EGFR	JAK-STAT, Notch, Dpp	Craig A. Micchelli	2011
LfcSCs	LFCR	Silent	Silent	Delta	-	-	Craig A. Micchelli	2011
ISCs	AM, PM	Active	Active	Delta, esg	Wg, JAK-STAT, etc.	Notch, JNK, etc.	Craig A. Micchelli Benjamin Ohlstein	2006 2006
HSCs	HPZ	Silent	Active	STAT	Wg, JAK-STAT	Hh	Shigeo Takashima	2008
RNSCs	Lower MTs	Active	Active	esg, JAK-STAT	JAK-STAT, EGFR, Scrib, Sav	JAK-STAT	Steven X. Hou	2007

#### Cellular constituents of the epithelium

As mentioned, six stem cell types exist along the adult gastrointestinal tract. The highly differentiated cells that are produced depend on the activities of these stem cells. For example, in contrast to vertebrates, in which, in addition to ISCs in mammals, the TA cells that occupy the crypt length also divide, the midgut epithelial cells of adult *Drosophila* are strictly postmitotic.

**Fig. 3:**
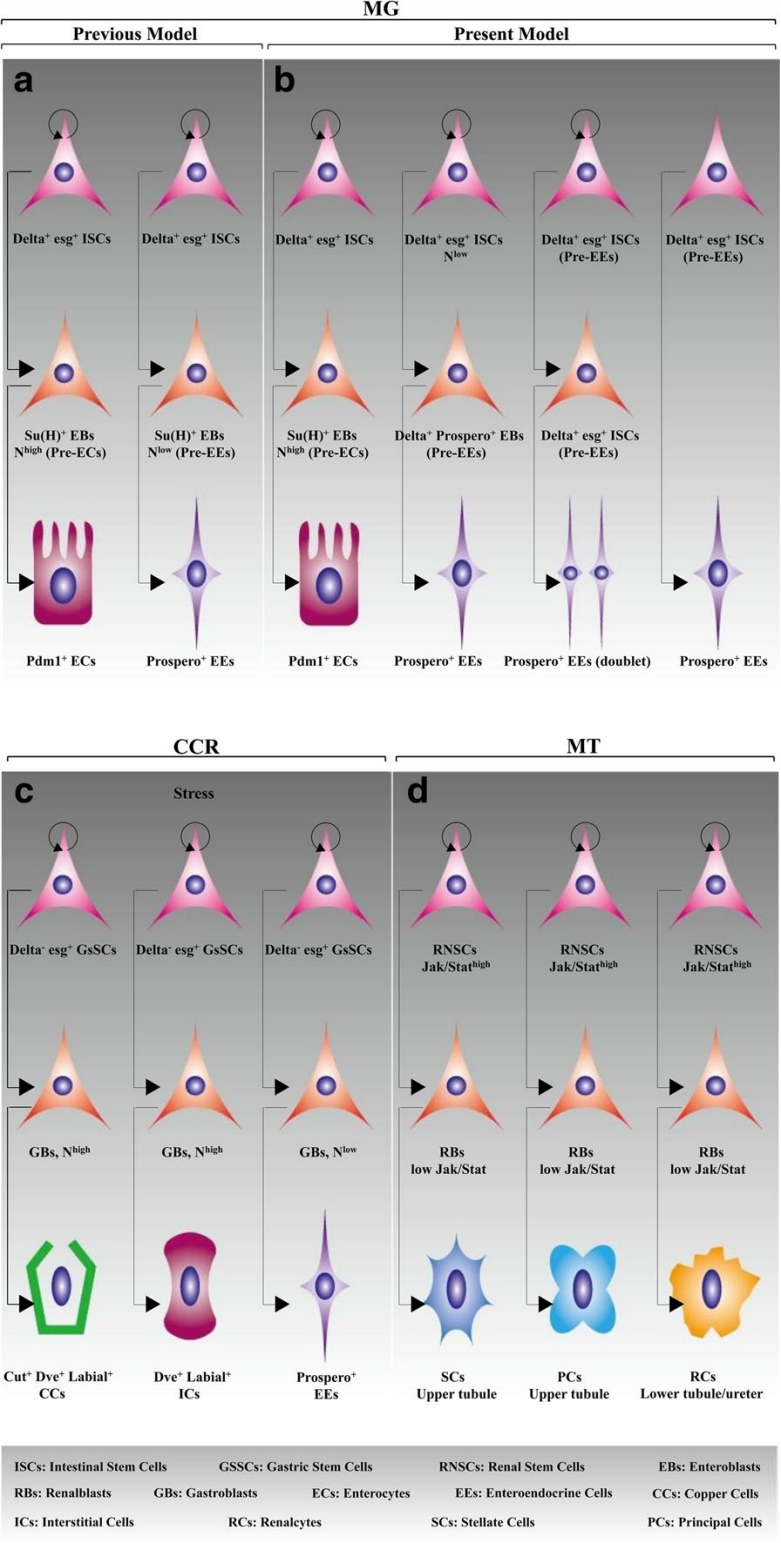
Developmental hierarchy. (**a**) Previous model of ISC division. ISCs and EBs are produced through the mitotic activity of Delta^+^ and esg^+^ ISCs. The EC and EE fates involve Delta/Notch. (**b**) New model of ISC division. The EE doublets are generated through ISC symmetric division. ISCs also become EEs directly. See text for details. (**c**) GsSC lineages. High-Notch GBs are committed progenitors for CC and IC differentiation. However, EEs are derived from low-Notch GBs. (**d** )RNSC lineages. The MTs consist of the SCs, PCs and RCs. These cells are produced by RNSCs through RBs

In this section, we first emphasize the cellular constituents of two common regions: the AM and the PM. Recent studies have facilitated rapid progress in understanding the organizational structure of these regions. They consist of a simple columnar epithelium surrounded by visceral muscle (VM), nerves and tracheae composed of several cell types, including ISCs and enteroblasts (EBs) as well as two functional cell types known as absorptive ECs and secretory enteroendocrine cells (EEs) [19, 37] (Fig. 2b and Fig. 3a).

How an ISC adapts to an EE fate is currently a topic of extensive debate. A new viewpoint was presented by Guo Z and Ohlstein B. After an ISC asymmetric division, two daughter cells are produced. One is a new ISC expressing Delta but with low Notch activity, and the other is a new EB expressing Delta and prospero. The latter is a Pre-EE that differentiates into a mature EE shortly thereafter [38]. This process is accompanied by Par complex-mediated prospero protein asymmetric division [38]. These findings have altered our perspective on EE production (Fig. 3b). Additionally, ISC symmetric division generates EE doublets, and an ISC may directly become an EE [39, 40]. However, to date, the exact mechanisms associated with these two additional EE production processes remain unknown. The *achaete-scute* complex (*AS-C*) genes, such as *scute* (*sc*) and *asense* (*Ase*), promote EE production but are not required for ISC maintenance [41]. Hou SX and colleagues identified a negative feedback mechanism responsible for EE production. EE-produced Slit interacts with Robo2 in ISCs. This Slit/Robo2 pathway in ISCs prevents new EE production [42]. Interestingly, the two EE subtypes known as class I and class II EEs are distinguishable based on the combinatorial expression of neuropeptide hormones, and Notch regulates class II EE establishment but not class I EE commitment [43].

Of all highly differentiated cells, ECs are the best understood. Generally, an ISC divides asymmetrically, generating two daughter cells. One is a new ISC expressing Delta, and the other is a new EB expressing Su(H) with high Notch activity. The latter is a Pre-EC that differentiates into a mature EC shortly thereafter. The high Notch levels in the new EB are responsible for EC fate determination, and this differentiation process is closely associated with the regulation of Delta/Notch [44]. Considering the aforementioned notion that each region is associated with a very specialized type of EC with various functions [28], these findings raise an important question regarding how this identity is determined during differentiation from EBs to ECs. Altogether, these findings support the idea that the basally located ISCs give rise to both ECs and EEs and that EBs are produced by ISC self-renewal. Importantly, the cellular constituents described above imply the manipulation of organismal recovery in response to exposure to internal and external factors (discussed hereafter).

We subsequently focused on the cellular constituents of other compartments, including the MM and MTs. The epithelium of the MM is functionally separated into three major domains: the CCR, the LFCR and the FCR. In the CCR, stress-induced GsSC activity is essential for the regeneration of all cell types, including acid-secreting copper cells (CCs), interstitial cells (ICs) and EEs [45]. These mature cells are generated through gastroblasts (GBs) produced by GsSCs. CCs and ICs are derived from common GBs expressing high levels of Notch; however, EEs may be indirectly derived from a separate GB population with low Notch activity (Fig. 3c). The LFCR and FCR are located at the posterior part of the MM [46, 47]. However, the roles of an unappreciated population of LFCs in the maintenance of MM physiological function remain unknown.

There have also been many exciting discoveries regarding the cellular constituents of MTs in recent years. RNSCs located in the lower tubules and ureters of the MTs are essential for the regeneration of injured renal tissue comprising stellate cells (SCs), principal cells (PCs) and renalcytes (RCs) (Fig. 3d) [36]. This observation has raised several important questions. For example, in addition to these three cell types, do any other types of differentiated cells exist in MTs? What are the functional contributions of RNSC-induced differentiated cells in the excretive process? Detailed descriptions of stem cell lineages are summarized in Fig. 3.

To summarize, understanding the complex, orchestrated activities of various signal transduction pathways significantly impacts our knowledge of the mechanistic details controlling GsSC or RNSC-mediated long-term homeostasis (discussed hereafter). This is of the utmost importance if we are to exploit the intricate set of networks involved in inflammation and tumorigenesis in other homeostatically growing tissues such as the human stomach and kidney. In the following sections, we review studies in adult *Drosophila* intestines that highlight the exact mechanisms by which an ISC instructs the process of intestinal regeneration and homeostasis, including those involved in ISC maintenance, proliferation and differentiation.

### Mechanisms facilitating the proliferative capability of ISCs

#### Mechanisms necessary to maintain ISC status

Data obtained from the well-studied *Drosophila* gastrointestinal tract model system have advanced our understanding of the conceptual framework that regulates the determination of stem cell fate. Recently, numerous studies have implicated a myriad of cellular signaling pathways in the regulation of ISC self-renewal properties [42, 48–53]. First, esg is a zinc-finger transcription factor that maintains stemness. According to Edgar BA and colleagues, suppression of esg in progenitors causes ISC state loss accompanied by an increase in Pdm1 levels [54]. Jones DL and colleagues also found that esg maintains stemness through the modulation of Notch levels. Loss of esg causes an increase in Amun, an inhibitor of Notch signaling, which contributes to an increase in the number of EEs [55]. Interestingly, a recent study revealed the importance of EB-expressed esg in the suppression of terminal differentiation [56]. Epithelial cell loss activates miR-8/miR-200 activity in EBs and suppresses esg activity through a reverse epithelial-mesenchymal transition (EMT) process. Decreased esg activity promotes terminal differentiation [56]. Together, these observations demonstrate that esg is of critical importance in regulating the maintenance of ISC status. Second, intriguingly, ISC maintenance relies on basal levels of the Janus kinase-signal transducer and activator of transcription (JAK-STAT) and Jun-N-terminal kinase (JNK) pathways [57, 58]. Lemaitre B and colleagues linked JAK-STAT pathway ligands to alterations in intestinal regeneration [57]. Specifically, Unpaired 1 (Upd1) in ISCs, a ligand of the JAK-STAT pathway that acts in an autocrine manner, regulates ISC maintenance under conventional conditions. However, Upd2 and Upd3 regulate ISC proliferation only in the aging intestine [57]. One of the most important questions that has arisen from the abovementioned findings is why different Upds regulate ISC behaviors during different stages. Third, the wingless (Wg) signaling pathway, an important ISC survival regulator, appears to promote stemness. The loss of Wg pathway components, such as Frizzled (Fz) and Fz2 or Armadillo, in ISCs results in the loss of ISC maintenance [59]. To summarize, these studies illustrate how classical signaling pathways, including the JAK-STAT and Wg pathways, as well as classical signaling through esg affect ISC maintenance.

In addition to the classical signaling pathways responsible for ISC maintenance described above, several other signaling pathways have been defined as effective regulators capable of maintaining stemness. First, integrin activity serves as a key regulator of the maintenance of ISC status. For example, according to Xi R and colleagues, the two α-integrin subunits αPS1 and αPS3 and the ß-integrin subunit mys are responsible for ISC maintenance [48]. Adachi-Yamada T and colleagues have also suggested that loss of the βν integrin triggers frequent ISC duplication through the depression of Notch signaling [49]. A similar result was obtained by Knoblich JA and colleagues, who suggested that knockdown of precursor-specific integrins leads to ectopic precursor clusters [60]. Second, Hou SX and colleagues identified four hundred and five genes that are responsible for ISCs [42]. For example, the knockdown of AurB and Dia leads to the development of larger ISC nuclei [42]. Likewise, the loss of Cep89 and borr blocks mitotic cell division and triggers excessive cell growth [42]. Signaling that regulates stem cell death and survival, such as that involving Arf79F and Garz from coat protein complex I (COPI), was also detected in this study [42]. Third, GATAe, the zinc-finger protein Charlatan (Chn) and Decapentaplegic (Dpp) are important regulators of ISC maintenance [51–53]. As shown by Adachi-Yamada T and colleagues, esg^ts^Gal4 > GATAe^IR^ intestines contain decreased numbers of Delta^+^ cells [51]. The regulation of normal chromatin structure requires Chn activity in precursors. esg^ts^Gal4 > Chn^IR^ intestines demonstrate decreased levels of H3K4me3, a chromatin marker involved in transcriptional initiation [52]. Additionally, the roles of bone morphogenetic protein (BMP) signaling in intestinal regeneration were revealed by Tian A and Jiang J, who suggested that knockdown of EC-specific Dpp triggers the loss of ISC maintenance [53]. Trachea-special Dpp is also responsible for ISC activity. Depletion of Dpp in tracheal cells activates ISC mitosis during homeostasis [61]. However, as shown in another study, the loss of trachea-expressed Dpp has no effect on ISC proliferative activity after damage, although VM-expressed Dpp is strongly associated with ISC division [62].

Additionally, we also discuss two fascinating studies that focused on ISC maintenance. For example, a wealth of data from Bardin AJ and colleagues supports the crucial role of Hairless in the inhibition of ISC loss [41]. Furthermore, Hairless-induced repression of the expression of the Notch target gene E(spl)-C is critical for maintaining stem cell fate and reducing Notch activation [41]. Daughterless (Da)-dependent bHLH activity caused by ISC-driven E(spl)-C inactivation is involved in ISC maintenance [41]. In contrast, in EBs exhibiting high levels of Notch activation, Da is inhibited by E(spl)-C, which downregulates the bHLH activity required for stem cell commitment, leading to the loss of stem cell characteristics [41]. Another example is from a study conducted by Xi R and colleagues. Target of rapamycin (TORC) activation, an additional determinant inhibited by tuberous sclerosis complex1/2 (TSC1/2), is essential for maintaining ISCs independent of nutritional status [63]. TORC hyperactivation is also associated with ISC loss [63]. These two findings support the idea that the loss of ISC maintenance appears to involve the dysfunction of Hairless and TORC. Interestingly, a recent study by Edgar BA and colleagues that focused on ISC pool maintenance suggested that ISC pools fail to recover after partial depletion in *Drosophila*, unlike in the mouse intestine, and ISC pools do not increase with infection. Differing requirements for ISC capability may be associated with differential ISC behavior between *Drosophila* and mice [64].

The field investigating sex differences in adult *Drosophila*, in particular, has witnessed significant developments in the past year; therefore, we will focus on this field in the following sections. Two recent studies have shed light on sex-biased concepts of stem cell behavior. For example, according to a report by Miguel-Aliaga I’s group, Sex lethal (Sxl) protein expression in female ISCs produces a female-specific isoform of transformer (tra), which, together with tra2, splices doublesex (dsx) to produce a female-specific form and splices fruitless (fru) to block production of a male-specific form in ISCs [65]. In contrast, Sxl expression in ISCs is not observed in males, and the male-specific isoform is present due to dsx and fru dysregulation in ISCs [65]. This study also suggested that the intrinsic sexual identity of ISCs contributes to the maintenance of organ size and the regulation of intestinal plasticity. For example, masculinization of ISCs led to a shorter midgut in females, which was similar to the midgut in males. This was largely due to a reduction in ISC proliferation in masculinized females [65]. Additionally, masculinized females were not capable of undergoing intestinal resizing after mating. The midguts of mated masculinized females had decreased Su(H)^+^ cell numbers compared with those of mated female controls [65]. Altogether, the signaling pathways associated with sexual identity may be highly complex. Considering that sexual identity in the nervous system was thought to be confined to fru- and dsx-expressing neurons [66], research investigating sex determinants in adult ISCs will advance our understanding of how sexual identity is regulated in other tissues/organs. Additionally, it is remarkable that the signaling pathways involved in intrinsic sexual identity in *Drosophila* and mammals include both the dsx/doublesex and mab-3 related transcription factor (Dmrt) family of transcription factors and their targets [67]. Therefore, data obtained from thorough studies investigating sex differences in *Drosophila* will facilitate the discovery of similar mechanisms in mammals, including humans. Further studies of sex differences have rapidly progressed, spurred by the recent discovery of substantial sexual dimorphism in aging flies [68]. Results from Partridge L and colleagues indicate that major intestinal deterioration occurs in aging females, which are more resistant than aging males after challenge due to higher levels of ISC division [68]. In contrast, the aging male gut, which demonstrates low ISC self-renewal activity, exhibits improved barrier function [68]. Additionally, feminized males exhibit increased ISC proliferation with age; however, this phenomenon does not occur in control males [68]. Regrettably, the effects of masculinization on female intestines have not been explained. Thus, we speculate that several remarkable changes might be observed in aging masculinized females, including decreased ISC division and weaker barrier function. A very important question regarding sex-biased concepts in the adult *Drosophila* gut remains for future studies. For example, Miguel-Aliaga I’s group implied an autocrine effect of ISC-expressed tra on the sexual identity of ISCs [65]. However, by mis-expressing tra in ECs in the male gut, Partridge L and colleagues found that males with feminized midguts develop female-like ISC proliferative activity during aging [68]. This raises the real possibility that key elements of sex-determination pathways in other types of intestinal cells (not only in ISCs) might also contribute to sex-biased concepts of ISC proliferative activity in a paracrine manner. This is a hypothesis that requires further investigation.

To summarize, this observation improves our understanding of sex differences in disease susceptibility and provides groundwork for research in the new field of sexual identity. There is an ongoing debate regarding whether other sex-determination pathways regulate sex differences in stem cell fate determination. Decisive conclusions will soon be reached based on the aforementioned evidence obtained from the perspective of sex differences.

#### Mitogenic signaling in response to environmental challenges

In this section, we first emphasize the switch between symmetric and asymmetric fate outcomes. There is growing recognition of variable stem cell division patterns [69–74]. In the canonical view, a new ISC and a nondividing EB are generated through asymmetric ISC self-renewal that appears to predominate and occurs throughout the majority of adulthood [75, 76]. Alternatively, ISCs or EB doublets originate from an ISC that divides symmetrically [69]. A novel signaling molecule that regulates symmetric ISC division, Lin-28, was recently revealed. In newly eclosed adults, the Lin-28-mediated outcome of symmetric stem cell division is a key determinant in establishing gut remodeling [69]. Sokol N and colleagues found that Lin-28 acts as an RNA-binding protein to interact with insulin receptor (InR) mRNA, regulating insulin levels in ISCs and contributing to ISC symmetric renewal. They also found that this process occurs independently of the microRNA let-7 [69].

In addition to the identification of a role for Lin-28 in determining symmetric fate outcomes, different groups have also recently assessed several novel signaling molecules that regulate asymmetric ISC division, including the Par complex and Sara endosome. For example, a study investigating asymmetric Par complex segregation during ISC division identified a critical role for integrin-dependent ISC polarity in asymmetric renewal [60]. Furthermore, during ISC mitosis, the mitotic spindle, which is involved in the fates of different daughter cells, is regulated by integrin-induced Par complex segregation. According to Gonzalez-Gaitan M and Montagne C, Notch bias during ISC asymmetric division is mediated by the ISC-specific Sara endosome, which is dispatched to the presumptive EB after mitosis [70]. Thus, all of these observations demonstrate that the Par complex and Sara endosome exert far-reaching effects on asymmetric renewal.

Additionally, although ISC division is morphologically symmetrical, ISC asymmetric division appears to be a fundamental consequence of the asymmetric segregation of Delta [77]. For example, the Delta protein is expressed in ISCs but not in EBs [44]. During ISC division, low levels of Delta protein inherited from ISCs may be degraded [78]. Altogether, we conclude that it is unclear how different fates arise, and little is known about the precise mechanisms responsible for ISC asymmetric division. Other mitotic events may be involved in the asymmetric outcome.

**Fig. 4:**
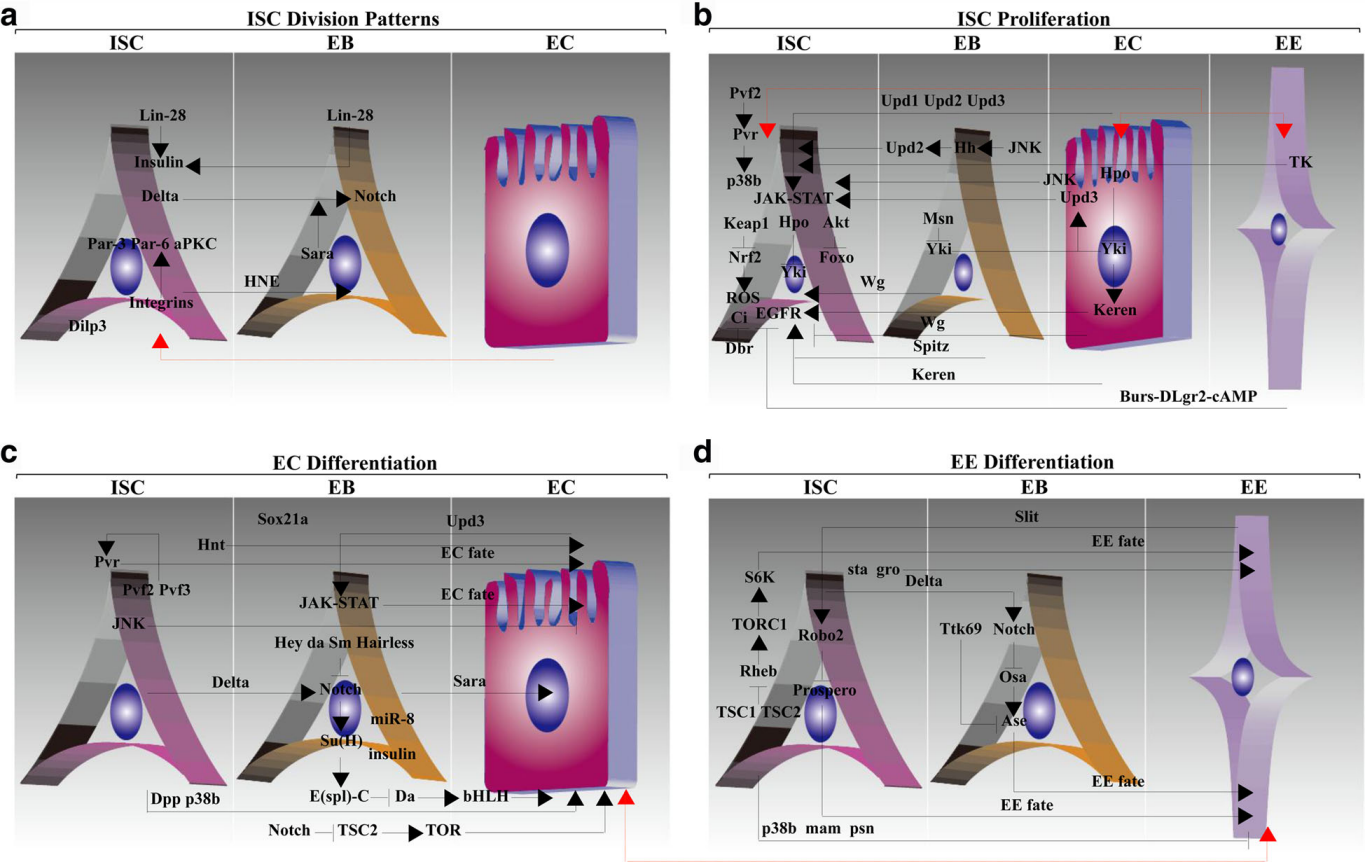
Signaling networks for the maintenance of homeostasis. **a** The factors that control ISC division patterns. (**b-d**) The signaling pathways that control ISC proliferation and differentiation. Many signaling cascades are crucial for ISC proliferative activity and EC specification. Several signaling cascades are associated with EE differentiation. See text for details. The red dotted lines in all the pictures indicate unknown

Finally, numerous studies have addressed how the pattern of ISC division changes in the aging intestine [3]. The predominance of symmetric division fates may contribute to the aging-induced increase in the ISC population. The deregulation of ISC proliferation is accompanied by the accumulation of mis-differentiated cells in the aging midgut [58]. Additionally, Yoo MA and colleagues have focused on age-related changes in the gut. Aging and oxidative stress increase Pvf2 activity, contributing to age-related increases in ISC number and activity [79]. Interestingly, in another study, Yoo MA’s group also explored the DNA damage response during aging. Two DNA damage response-related signaling pathways, ataxia telangiectasia-mutated (ATM) and ATM- and RAD3-related (ATR) kinases, were responsible for ISC maintenance and proliferation. ATM and ATR activation were increased with age, and ATM and/or ATR loss in precursors reduced aging-induced ISC proliferative activity [80]. Importantly, identification of the molecular mechanisms that mediate symmetric ISC division in the aging intestine will provide a platform to improve certain aspects of specific aging-related diseases in humans. Detailed descriptions of signaling molecules involved in ISC division patterns are summarized in Fig. 4a.

To summarize, tissue maintenance for long-term homeostasis is frequently associated with two stem cell fate patterns, and strict symmetric ISC division in *Drosophila* is the emerging hypothesis to explain how tissue turnover is achieved by multifunctional stem cells.

Constant turnover requires multiple pathways that converge on shared targets following exposure to numerous insults [16, 81]. Next, we will focus on depicting a high-resolution picture of mitogenic signaling in ISCs and discuss in detail several classic regulatory networks that are employed by ISCs to ensure appropriate proliferative activity and tissue homeostasis (Fig. 4b). Although an exhaustive summary of all the data in this field would be impossible, this review will highlight emerging trends.

A recent expansion of the literature has focused on the epidermal growth factor receptor (EGFR) pathway, one of the most highly modulated pathways in intestinal homeostasis, because this pathway is thought to be responsible for cellular behaviors ranging from cell growth and regeneration to survival in both *Drosophila* and mammals. For example, EGFR signaling promotes early tumorous ISC proliferation or differentiation-defective ISC-like cell proliferation [82, 83]. VM-specific vein (Vn), an EGFR ligand, activates the EGFR signaling pathway in ISCs to regulate ISC self-renewal, and midguts lacking Vn in the VM exhibit decreased ISC proliferation and an overall decline in tissue regenerative potential [84–86]. Additionally, Spitz (Spi) and Keren (Krn), two EGFR ligands, are essential for ISC division via the EGFR/RAS/RAF/mitogen-activated protein kinase (MAPK) pathway after infection [85]. Damaged ECs release Upd3, leading to Vn ligand production from the VMs, a process that is dependent on STAT activation in VMs in response to infection [84]. Loss of EGFR in ISCs inhibits ISC division triggered by ectopic Upd, supporting communication between the JAK-STAT and EGFR pathways to regulate ISC proliferation [84, 85]. Altogether, these observations demonstrate that the ligands Vn, Spi and Krn are important regulators that control ISC division, and ISC-derived EGFR activity is strongly associated with intestinal homeostasis. Interestingly, according to Lemaitre B and colleagues, EC-specific EGFR pathway activity is responsible for gut morphogenesis and the delamination and anoikis of damaged ECs [84]. EGFR-mediated extracellular signal-regulated kinase (ERK) activation is associated with Capicua (Cic) localization in the cytoplasm in response to stress, which causes ISC division via string (stg), Cyclin E (CycE), Ets21C and Pointed (Pnt) transcription triggered by Cic repression [87]. Importantly, several signaling pathways that inhibit EGFR activity to control ISC division have also been explored. For example, the silencing of several negative regulators of the EGFR signaling pathway, including *Cbl*, *Kek3*, *ttk*, *Cic*, and *CG15528*, leads to midgut hyperplasia, as confirmed by a recent screen of 44.8% of the *Drosophila* genome [42]. We conclude that the mechanistic details of EGFR-mediated ISC activity are likely to be quite complex. Future studies will focus on exploring the links between EGFR and other signaling pathways. We next discuss an interesting study that investigated how EEs and ISCs communicate with each other. Inter-cell communication between ISCs and mature differentiated cells was recently elucidated in a study investigating the role of EEs in the modulation of ISC behavior [86]. VM-derived Vn ligand suppression was associated with normal levels of the neuroendocrine hormone Bursicon (Burs) [86]. Loss of EE-derived Burs stimulated Vn/EGF activity, contributing to a mild increase in ISC proliferation [86]. Thus, Vn/EGF serves as a crucial node in the EE-ISC communication signaling network that governs ISC proliferation. Recently, alterations to AMP levels following EE loss were investigated by Buchon N and colleagues, who tested susceptibility to bacterial infection in flies with Prospero^+^ cell ablation from the gut to demonstrate the importance of EEs in responses to pathogens [18]. Considering the altered proliferative activity of ISCs upon EE loss described above, we speculate that the low survival of Prospero-insufficient flies discussed by Buchon N and colleagues may be attributable not only to decreased AMP levels but also loss of the stem cell-mediated steady state.

Research investigating the involvement of the JAK-STAT pathway in ISC activity, similar to that of the EGFR pathway, has been fruitful. The JAK-STAT pathway, as one of the major eukaryotic cascades, is conserved across phyla. However, numerous studies investigating the JAK-STAT network have yet to unravel the roles of several confirmed elements in ISC division [21, 81]. For example, the JAK-STAT pathway was implicated in homeostasis through the activation of the Upd cytokines induced by the oxidative burst and by damaged ECs [15, 88]. The ISC-specific JAK-STAT pathway affects ISC proliferation, but differentiation is regulated by this pathway in EBs. The mitotic response of ISCs is strongly suppressed by EC-specific Upd loss, allowing flies to succumb to infection [57, 88, 89]. The above findings suggest that EC-specific Upd cytokines play an important role in activating the JAK-STAT network and contribute to ISC self-renewal. Notch signaling was recently shown to interact with the JAK-STAT pathway. Hou SX and colleagues identified the role of Notch signaling in suppressing ISC proliferation via the JAK-STAT pathway [90]. Stat92E was predominantly localized within the nuclei in Notch mutant clones, suggesting that loss of Notch elevated JAK-STAT levels. Furthermore, Upd activity was induced in ISC-like clusters where Notch was inhibited. Thus, Notch appears to regulate JAK-STAT levels by suppressing Upd transcription. We subsequently discuss the studies conducted by Xi R and colleagues investigating the roles of the JAK-STAT signaling pathway in ISC activity. First, their recent study examining ISC self-renewal and differentiation has provided a key clue to answer the question of how Upd from the muscular niche controls JAK-STAT levels in ISCs to regulate ISC activity [91]. This study also suggested that the JAK-STAT pathway functions upstream of Notch, in parallel with Wg, to control ISC self-renewal [91]. Second, remarkably, the EGFR, Wg and JAK-STAT signaling pathways appear to be integrated to regulate ISC activity. This was confirmed by an interesting study suggesting that the loss of any single one of the three signaling pathways caused mild ISC loss, while suppressing all three pathways triggered complete ISC elimination [92]. Additionally, JAK-STAT hyperactivation is capable of promoting ISC division in the intestines with EGFR loss [92]. Thus, like Notch, EGFR and Wg also communicate with the JAK-STAT pathway to regulate ISC activity. Altogether, to maintain intestinal homeostasis, a link between JAK-STAT and other signaling pathways is required. If these signaling networks break, deficiencies in ISC self-renewal may occur. Interestingly, hemocyte-expressed Upds activate the JAK-STAT pathway in the gut, regulating ISC mitogenesis upon injury. This finding suggests that hemocyte-gut communication acts as a critical strategy to regulate the ISC response [93].

The insulin pathway acts as a conserved nutrient-sensing regulator, coupling dietary conditions with the control of tissue growth in metazoans. The activation of ISC division and growth also requires InR and several downstream effectors [81]. For example, according to Ohlstein B and colleagues, decreased ISC proliferation is associated with contact between ISCs and their descendants due to the nutrient deprivation-mediated dysfunction of insulin [94]. Nutrient deprivation and reduced insulin levels result in prolonged contact between ISCs and newly formed daughters, contributing to ISC proliferation defects. Increased ISC division is apparent following loss of the cell adhesion molecule E-cadherin (E-cad) [94]. This finding suggests that insulin-mediated proper contact between precursors is responsible for ISC proliferative activity. Intriguingly, the nutrition-mediated insulin signaling pathway in ISCs induced by the VM-specific insulin growth factor-like peptide 3 (Ilp3) drives midgut growth, and a switch from asymmetric to symmetric division is associated with organ resizing [76, 95]. Altogether, unlike the EGFR and JAK-STAT pathways, the functional impacts of the insulin pathway in ISC self-renewal are still poorly understood. As mentioned above, Lin-28 interacts with InR mRNA to regulate insulin levels in ISCs, controlling ISC division [69]. Thus, better understanding insulin-mediated intestinal homeostasis promises to be an exciting area of study.

Dysregulation of Wg signaling leads to colorectal cancers in humans, and the importance of the Wg pathway as a pivotal regulator of ISC mitosis in *Drosophila* is now being explored. For example, Wg is expressed in a small band of VM cells. VM-specific Wg cooperates with downstream components, such as Fz, Dishevelled and Armadillo, to maintain ISC proliferation [59, 96, 97]. A higher rate of ISC proliferation is observed in the intestine as a result of the loss of *dAxin* or *adenomatous polyposis coli* (*Apc*) in ISCs, resulting in Wg activation [98]. Similarly, the overexpression of Armadillo, an active form of β-catenin, triggers excessive ISC proliferation activity [44, 59, 99]. Like Upds, massive ISC division and hyperplasia are also induced by Wg overexpression [59]. The Wg cascade in ISCs is stimulated by VM-specific Wg, resulting in ISC division [59]. These studies suggest that VM-specific Wg is sensed by the ISCs and that it controls their self-renewal. However, the role of VM-specific Wg in intestinal homeostasis and regeneration is extremely controversial. For example, Sansom OJ and colleagues found that Wg in VM is not required for ISC function because loss of Wg in VM triggers no change in ISC activity after damage [100]. Interestingly, Wg in EBs is strongly induced after damage. Furthermore, loss of EB-derived Wg causes a decrease in ISC proliferation [100]. Altogether, these studies suggest that Wg acts as a critical regulator that controls ISC activity under normal conditions and after damage. Importantly, the functions of the Wg, EGFR and JAK-STAT pathways in ISC division and epithelial self-renewal in *Drosophila* are relevant to our understanding of several human genetic diseases, including chronic inflammatory disorders and cancers. In mammals, the Wingless and INT-1 (Wnt) and EGFR pathways are responsible for ISC division [101]. Loss of Wnt effectively decreases ISC proliferative activity in the crypts, destroying the intestinal epithelium [102, 103]. Interestingly, Paneth cells produce growth factors, such as the EGF and Wnt ligands, that are strongly associated with ISC proliferation and maintenance [104]. Additionally, in a mouse model of damage–induced colitis, Karin M found that STAT 3 is responsible for ISC proliferation [105]. Consistently, the loss of suppressor of cytokine signaling 3 (SOCS3), the STAT inhibitor, increases the proliferative response to damage and inflammation-associated colon tumorigenesis [106].

A unique epithelial oxidative burst in the adult *Drosophila* intestine is responsible for antimicrobial responses. Surprisingly, the role of oxidative stress in ISC mitosis is of particular interest. For example, according to Jasper H and colleagues, low levels of ROS in the intestines are maintained indirectly by Nrf2 and its negative regulator Kelch-like ECH-associated protein 1 (Keap1) [107]. Damage-induced intestines with Nrf2 loss triggered by Keap1 overexpression exhibit increased ISC proliferative activity. This is due to the inactivation of Nrf2-mediated anti-oxidative genes and high ROS levels [107]. This finding suggests that Nrf2 and Keap1 serve as two crucial nodes in a redox control mechanism that governs ISC proliferative activity. Despite its remarkable role in ISC activity, the nature of the interaction between these signals associated with ROS levels is still poorly understood. Interestingly, a recent study provided a link between oxidative stress and mitogenic signals: Perrimon N and colleagues found that transient receptor potential A1 (TRPA1) and ryanodine receptor (RyR) are the two regulators of cytosolic Ca^2+^ levels in ISCs. During homeostasis, basal levels of ROS activate TRPA1 and RyR activity in ISCs, contributing to low levels of cytosolic Ca^2+^. This activation is responsible for Ras/MAPK activity, causing ISC proliferation. Based on this finding, TRPA1, RyR, cytosolic Ca^2+^ levels and the Ras/MAPK pathway in ISCs are important for ROS-induced ISC division [108]. Additionally, Lemaitre B and colleagues found that dual oxidase (DUOX)-mediated oxidative bursts are responsible for ISC proliferation. Loss of DUOX leads to decreased ISC proliferative activity after infection [88]. However, an important question remains for future studies: do the exact mechanisms identified to date that control ROS levels in ECs (DUOX-mediated ROS generation) also regulate oxidative stress in ISCs?

Next, this review will focus on two other classic regulatory networks: the JNK and Hippo pathways. First, the JNK pathway, which is triggered by a variety of stress signals, promotes ISC proliferation by inducing the expression of ligands of the JAK-STAT and EGFR pathways [15, 17]. Additionally, activation of the JNK pathway in aging flies results in ISC expansion and the accumulation of mis-differentiated cells [15, 58, 88]. Compensatory ISC proliferation is a consequence of EC infection-induced elimination caused by the activation of JNK in differentiated cells [58]. These observations illustrate that the JNK pathway is critical to ensure proper ISC responses to stress conditions, including aging and pathogen infection.

Second, the Hippo pathway, which was discovered in genetic screens of the *Drosophila* eye [109], has previously been implicated in the regulation of tissue growth and apoptosis. Research investigating the roles of the Hippo pathway in intestinal regeneration has clearly progressed over the past few years. For example, Hippo and Warts (Wts) inactivation-induced Yorkie (Yki) expression is required for ISC proliferation through the JAK-STAT pathway in response to tissue damage [110–113]. EC-specific Hippo pathway inactivation is responsible for dramatic ISC over-proliferation [110]. The loss of Hippo in precursors also stimulates ISC proliferation [113, 114]. Hippo and Yki are required precursors for dextran sulfate sodium (DSS)-induced ISC proliferation [110]. Thus, these findings suggest that Hippo acts as a negative regulator to control ISC division. To summarize, although our understanding of the functional roles of these two pathways in intestinal regeneration has expanded considerably, as for the insulin pathway discussed above, important questions remain to be addressed: how is the JNK pathway activated in the gut after damage? What are the functional differences of the JNK pathway under different stress conditions, such as aging and oral infection? Are there other signaling pathways that interact with the Hippo pathway to influence intestinal homeostasis?

Finally, an additional level of complexity has recently been added to this picture with the identification of several additional signaling cascades involved in the regulation of ISC mitosis. Initially, we provide several interesting findings with respect to the roles of several mitogenic signaling molecules during two particular adult stages: aging and mating. For example, the PDGF/VEGF receptor (PVR) was reported to regulate ISC proliferation in response to oxidative stress [79, 115]. In aging flies, the PVR ligand Pvf2 promotes increased ISC division [79]. The proliferative activity of ISCs is governed by the dynamic regulation of intracellular Ca2^+^ levels [116], an idea supported by Jasper H and colleagues. This group also suggested that lifespan is limited by the activity of the aging-associated PKR-like ER kinase (PERK), which induces ISC hyperproliferation in response to endoplasmic reticulum (ER) stress [117]. A recent study by Jones DL and colleagues has suggested that the mitophagy-related signalings phosphatase and tensin homologue-induced putative kinase 1 (Pink1) and Parkin are associated with ISC proliferation during stress and aging. Precursor-specific knockdown of Pink1 or Parkin alters mitochondrial morphology and density, and leads to the reduction of age-induced ISC proliferation, contributing to the maintenance of intestinal homeostasis [118]. Interestingly, a remarkable discovery in the aging intestine by Walker DW and colleagues suggested that the age-dependent loss of intestinal integrity contributes to the pathophysiology of aging. Age-related increases in AMP expression are linked to intestinal barrier dysfunction, contributing to altered metabolic homeostasis and spontaneous physical activity [119]. Altogether, studies using the aging intestine model have led to significant advances in the elucidation of the functions of mitogenic signaling molecules. Further analyses using the aging intestine model will be expected to facilitate our understanding of the molecular mechanisms underlying age-related changes in stem cell behavior in mammals. Additionally, exploring the factors responsible for ISC activity after mating has been a recent focus of interest. According to Miguel-Aliaga I and colleagues, anticipatory endocrine signaling via juvenile hormone (JH) after mating is associated with ISC activity and organ growth in females, and this is regulated by Methoprene-tolerant (Met) and Germ cell-expressed (Gce) [120]. However, many questions remain. For example, are any other hormones responsible for this intestinal remodeling after mating? If so, the next big challenge will be to understand how these hormones are coordinated to achieve intestinal remodeling.

Next, we present two recent studies of the roles of the extracellular matrix (ECM) in intestinal homeostasis. First, Perlecan (Pcan) is a highly conserved ECM component, and ISC ECM attachment triggered by Pcan is crucially important for regulating ISC proliferation [121]. Lin X and colleagues found that ISC identity and proliferative activity are clearly disturbed when they inhibit Pcan activity in ISCs (but not in other surrounding cells) [121]. This result was not due to the inhibition of EGFR and JAK-STAT, but integrin activity was an important factor for the regulation of Pcan-mediated ISC function [121]. Second, another novel regulator of ISC proliferation, heparin sulfate 3-O sulfotransferase A (Hs3st-A), was also identified by Lin X and colleagues. Hs3st-A and Hs3st-B are important regulators of heparan sulfate biosynthesis, which is associated with ECM production. The loss of EC-specific Hs3st-A causes ISC hyperproliferation due to increased EGFR levels [122]. ISC hyperproliferation also occurs in the intestines with Hs3st-B-insufficient ECs [122]. Altogether, this series of remarkable results suggests that Pcan, Hs3st-A, and Hs3st-B play roles in controlling ISC proliferative activity, and the ECM regulates intestinal homeostasis. Additionally, this group demonstrated the association of sterile-like 20 kinase, Tao, loss-induced rapid ISC proliferation with Hippo pathway activation, which is accompanied by an increase in JAK-STAT pathway activity levels [123].

Finally, we focus on several additional regulators of ISC proliferation, including Sox21a, bantam, Src non-receptor kinases and the zinc-finger protein Chn. For example, Biteau B and Meng FW reported the contribution of Sox21a protein induction mediated by the JNK and ERK pathways following damage to ISC proliferation [124]. Interestingly, Lemaitre B and colleagues identified a feed-forward loop associated with EB accumulation and tumor formation. Loss of Sox21a leads to EB accumulation. Accumulating EBs release Upd2, which activates ISC proliferative activity. Increased ISC division is responsible for Sox21a tumor initiation and growth [125]. Furthermore, Zhang L and colleagues have provided fascinating new insights into the functional role of bantam, specifically revealing the relationship between precursor-specific bantam and pathogen-induced ISC division [126]. These findings suggest that Sox21a and bantam are novel contributors to the damage-induced regulation of ISC activity. Events late in colonic tumor progression appear to be regulated by the Src non-receptor kinases [127]. Edgar BA and colleagues found that the accumulation of division-capable EB-like cells is triggered by Src42a and Src64b level-induced G1/S and G2/M cell cycle phase progression, which causes excessive ISC division [128]. The roles of zinc-finger proteins in ISC proliferative activity remain enigmatic. Recently, Ip YT and colleagues identified the zinc-finger protein Chn, which is associated with gut homeostasis. Flies lacking Chn protein activity demonstrate regeneration failure [52]. The above examples suggest that the Src non-receptor kinases and the zinc-finger protein Chn are critically important for regulating ISC activity. Additionally, the Misshapen-Wts-Yki pathway in EBs is also essential to achieve intestinal homeostasis by regulating ISC division [129].

To summarize, given the aforementioned description, it is tempting to speculate that mitogenic signals capable of remodeling the midgut epithelium are likely to be just as diverse. Importantly, certain features of the basic mechanisms regulating tumorigenesis in humans will be elucidated by applying tools to examine the adult *Drosophila* gastrointestinal tract given the increasingly sophisticated characterization of epithelial carcinogenesis and the conservation of certain stem cell division responses across species. Several drugs can be mixed with *Drosophila* food, and the technology for feeding *Drosophila* with these mixtures is mature. Therefore, it should be possible to use *Drosophila* tumor models to screen for drugs [130]. Before 2013, data from large-scale chemical screens using adult *Drosophila* were lacking [131]. However, a systematic screen of chemotherapeutics in a *Drosophila* intestinal tumor model was reported one year later. For example, chemotherapeutics can inhibit the growth of tumor stem cells. Using a tumor model in the adult *Drosophila* gut, Perrimon N and colleagues found that several chemotherapy drugs also paradoxically promote ISC hyperproliferation, contributing to tumor recurrence. Given that the evolutionarily conserved JAK-STAT pathway is associated with chemotherapeutics-induced tumor recurrence, a similar effect may occur in humans [132]. Additionally, as mentioned above, Arf79F from COPI is responsible for ISC survival [42]. Recently, Hou SX and colleagues suggested that normal or cancer stem cells may rely primarily on lipid reserves for energy. The loss of Arf79F in ISCs kills normal and transformed stem cells through necrosis. This process is achieved by attenuating the lipolysis pathway. Importantly, Arf79F inhibitors are strongly associated with a decrease in cancer stem cells in human cancer cell lines. This breakthrough discovery implies that targeting the COPI complex or the lipolysis pathway may represent a novel approach for cancer therapy in human [133].

#### Inducers capable of provoking differentiation

Thus far, we have focused on the proliferative activity of ISCs. ISC renewal and differentiation work together to promote mucosal homeostasis; however, how intestinal cell differentiation and epithelial renewal are stimulated has not yet been fully characterized. In this section, we discuss recent evidence supporting the involvement of several determinants in the initiation of ISC differentiation.

(1) In response to Gram-negative bacterial infection, DUOX-medicated ROS generation facilitates the rapid regeneration of the compromised intestine via the production of new mature differentiated cells. This stress-mediated differentiation activity occurs independent of intestinal cell apoptosis [134]. Thus, we conclude that oxidative stress acts as an important inducer of intestinal differentiation. (2) The ingestion of DSS and bleomycin triggers epithelial cell loss accompanied by an increase in the rate of ISC division, which leads to increased levels of EC differentiation via newly divided EBs [62, 135]. The differentiation response to apoptosis is not only caused by chemicals. Intestines infected orally with several microbial species, such as enteric pathogens, also exhibit increased levels of differentiation [17, 89]. Data presented by Miura M and colleagues suggest the ability of ROS to stimulate the apoptotic caspase pathway after systemic responses to wounding. Intestines lacking caspase pathway activity exhibit defects in homeostatic regeneration [136]. These findings indicate that apoptosis provokes intestinal differentiation in response to chemicals and pathogen infection. (3) Considering the existence of peptidoglycan (PGN) released from symbiotic bacteria and pathogens under conventional conditions, which plays a fundamental role in regulating AMP synthesis and production, we speculate that, like ROS, additional factors, such as AMP levels in the daily environment, are also likely to stimulate the behavior of differentiation; this is an idea that requires further investigation [15, 137]. In the following sections, we review emerging data supporting the existence of precise mechanisms associated with the process of differentiation, including EC and EE production.

### Regulation of differentiation

In principle, to maintain continuous cellular turnover and intestinal regeneration in response to diverse types of gut epithelial injury, it is necessary to balance the proportion of stem cells with their differentiated descendants to maintain a “steady state”. Here, we review the current literature describing the features of terminal differentiation responsible for the production of highly differentiated cells.

#### Programs underlying the commitment of ISCs to discrete lineages

First, we emphasize the programs responsible for EC fate determination. Overall, early mechanistic studies of the differentiation programs focused almost exclusively on EC specification. Therefore, our current knowledge of differentiation-promoting factors that regulate EC specification is relatively complete (Fig. 4c). Initially, we provide several findings from an important study conducted by Hou SX and colleagues. This study identified ninety-eight genes that were found to be essential for the premature differentiation of ISCs into ECs [42]. For example, the knockdown of *Simj* and *Caf1* triggers a defect in the differentiation capacity of ISCs for ECs [42]. This group also indicated several signaling molecules that inhibit Notch activity. It is noted that ISC-specific Notch activation is associated with the suppression of ISC proliferation. The differentiation process is triggered by Notch in EBs and is mediated by the Delta protein from ISCs [138, 139]. According to Hou SX and colleagues, Hey, da, and Smr are negative regulators of Notch that affect ISC-to-EC differentiation [42]. These findings suggest that, unlike the *Simj* and *Caf1* genes, signaling molecules that inhibit Notch activity act as negative regulators of EC differentiation. Additionally, several gene products that function in the RNA polymerase II transcription cofactor mediator complex are also essential for EC differentiation [42]. The results obtained from a systematic genome-wide RNAi screen also indicate an association between gpp/dDot1 and the production of mature ECs [42]; knockdown of this signaling led to premature ISC-to-EC differentiation. Similar functions for Uba1 and ken, which regulate Ras and the JAK-STAT pathway, respectively, were confirmed in the same study. This study showed that the knockdown of Uba1 and ken resulted in increases in the number of ECs [42]. This series of remarkable results suggest that, like Hey, da, and Smr discussed above, gpp/dDot1, Uba1 and ken are also negative regulators of EC differentiation. Altogether, the results of this important study suggest that the question of which signaling pathways regulate EC differentiation may be of great complexity.

In addition to the signaling molecules mentioned above, additional EC differentiation-promoting factors have been explored recently. For example, as mentioned above, the zinc-finger protein Chn and the PVR signal transduction pathway play important roles in ISC maintenance and proliferation, respectively. Chn has also been identified as an essential regulator of differentiation [52]. Ip YT and colleagues found that overexpression of Chn blocks EC differentiation. Interestingly, Chn loss resulted in normal levels of EC differentiation. Likewise, the PVR signal transduction pathway is also responsible for differentiation, and ISCs containing alterations in the Pvf/PVR pathway exhibit defective EC differentiation [79]. These findings suggest that Chn and Pvf/PVR are strongly associated with ISC actions, including maintenance, proliferation and differentiation. Dpp is linked to multiple aspects of development in the *Drosophila* gastrointestinal tract, similar to its role in mammals. According to Boutros M and colleagues, the EB-specific loss of Dpp causes ISC hyperproliferation and defective EC maturation under normal conditions or in response to oral infection [140]. Importantly, Dpp in the CCR is also fundamental for CC differentiation [141]. Additionally, the p38 MAPK pathway appears to be induced by physical and chemical stresses in mammals. Yoo MA and colleagues revealed an association between oxidative stress-induced EC mis-differentiation and the p38b MAPK pathway in aging flies [142]. The phenotype involving oxidative stress-mediated large esg^+^ and Su(H)GBE^+^ cell expansion observed in the intestines of wild-type flies was not present in intestines expressing D-p38b^as^ in precursors [142]. This observation indicates that p38b activity in response to oxidative stress results in defects in precursor expansion and EC differentiation. Interestingly, a recent study by Edgar BA and colleagues highlighted the importance of postmitotic EC growth after damage, suggesting that EGFR/MAPK is very important for damage-induced EC endoreplication, and both EGFR/MAPK and Ras/MAPK upregulate E2f1 levels to induce EB/EC growth [143]. Finally, Sox21a was described in the preceding text as an important factor regulating ISC proliferation. According to Xi R and colleagues, Sox21a from EBs is also associated with EC differentiation [144]. Furthermore, Sox21a overexpression had no impact on ISC-expressed Pdm1 levels but led to an increase in Pdm1 levels in EBs. This finding suggests that EB-EC differentiation, but not ISC differentiation, is regulated by Sox21a activity. Importantly, a recent study by Lemaitre B and colleagues has suggested that Sox21a promotes differentiation by enhancing the Delta/Notch and the JAK-STAT-Sox21a-GATAe signaling pathway is responsible for the process of EB to EC differentiation [145]. Altogether, combined with the previous discussion, these data substantiate the idea that Sox21a not only is responsible for ISC proliferation but also regulates EB-EC differentiation.

One important reason that the field investigating the intestinal endocrine system gained much more attention and respect is that the endocrine functions of EEs correlate with multiple aspects of physiology and metabolism. Thus, this review next summarizes several studies that investigated well-established regulators acting as dedicated factors facilitating commitment to the endocrine lineage.

Recent studies examining homeostasis have begun to shed light on the regulation of EE fate, which is a relatively new field of study of highly differentiated cells (Fig. 4d). Whether EE lineages are derived from committed progenitor intermediates is currently the subject of extensive debate. Cutting-edge descriptions of EE fate determination suggest that EE commitment is established in ISCs rather than in EBs [39]. Subsequently, we will primarily discuss three key regulators of EE differentiation, including the Osa-containing complex, the transcriptional repressor Ttk69 and the Slit/Robo pathway. (1) The Osa-containing complex plays well-described roles in EE fate determination [146]. For example, evidence from Hou SX and colleagues has demonstrated the involvement of a differentiated progeny population in determining levels of the Osa-containing complex, the loss of which causes a decrease in the EE population [146]. Intriguingly, the Osa protein regulates EE differentiation depending on the activity of the transcription factor Ase [146]. Thus, this important finding reveals a remarkable role for Osa and Ase in EE differentiation. However, much remains to be learned about these molecular interactions. (2) Approximately two years later, Xi R and colleagues found that the BTB domain-containing transcriptional repressor Ttk69 is also crucial for the regulation of EE differentiation [147]. The derepression of sc and Ase, two components of the AS-C, is essential for precursor-specific Ttk69 depletion, which triggers an expansion of the ISC population and increases the number of EEs [147]. Furthermore, precursors forced to express Ttk69 do not adopt EE specification, and EE expansion in the intestine of Ttk69-depleted precursors is not inhibited by Notch overexpression, supporting the absence of a connection between Ttk69 and Notch [147]. This observation suggests that, like Osa, Ttk69 also interacts with Ase to regulate EE differentiation. However, one difference between the two signaling molecules is that Osa functions downstream of Notch to regulate EE differentiation [146], while there may be no link between Ttk69 and Notch. (3) Slit, involving three Roundabout receptors, Robo1–3, is responsible for regulating several fascinating aspects of *Drosophila* biology. Negative feedback control of EE fate determination has been identified by Hou SX and colleagues and by Jasper H and Biteau B [40, 42]. Hou SX and colleagues have indicated that the Slit/Robo pathway in ISCs, either upstream of or in parallel with the AS-C, is responsible for EE fate commitment [42]. In addition, prior to the establishment of differential Notch levels, ISC-specific suppression of Prospero protein expression is achieved via Robo2 activity to mediate EE specification [40]. However, recent data from Bardin AJ and colleagues argues against the existence of local feedback signals, instead identifying Numb as a crucial factor for EE fate. Furthermore, Numb triggers EE differentiation by inhibiting Notch activity [148]. Finally, in addition to the three main EE differentiation-promoting factors mentioned above, Hou SX and colleagues also identified sixteen other genes regulating EE differentiation. For example, eIF4H, a translation initiation factor, is closely linked to ISC-to-EE differentiation [42].

Notably, there is now substantial evidence suggesting the activity of several pathways is somewhat surprising given their impact on both EC and EE fate determination. For example, according to Jasper H and colleagues, Notch-mediated TSC2 repression in EBs regulated EC fate determination [50]. The commitment of EBs to the EE fate also required TSC activity [50]. Additionally, according to Adachi-Yamada T and colleagues, the GATA factor GATAe, which is required for EC and EE differentiation, is of special interest [51]. Thus, considering the previous discussion, we suggest that GATAe is associated with multiple aspects of intestinal homeostasis, including ISC maintenance, EC differentiation, and EE differentiation. A functional role for cyclin-dependent kinase 4 (Cdk4) in intestinal turnover was suggested following the recent discovery that defects in EB differentiation are triggered by the loss of Cdk4 activity, leading to a decrease in both EC and EE numbers [149]. Thus, these three findings indicate that TSC, GATAe and Cdk4 are responsible for both EC and EE differentiation. These studies raise an interesting question: why are these factors capable of regulating both highly differentiated cell fate determinations?

To summarize, how ISCs grow to manipulate lineage commitment during intestinal regeneration remains unknown, and signaling mediating the differentiation process is not exclusively confined to the above-mentioned regulators. Despite comprehensive knowledge of terminal EC differentiation, other factors remain to be elucidated. Additionally, our attempts to further characterize the molecular basis of EE fate establishment are currently underway. Given that considerably less is known about EE differentiation, evidence uncovering the precise mechanisms that regulate EE differentiation should be available in the near future. Finally, the precise molecular cascades underlying the communication between ECs and EEs remain unclear. In the remaining text, we will outline the basic research areas regarding several mechanisms involved in the maintenance of homeostatic equilibrium by other stem cell types.

### Other types of stem cell-mediated homeostasis

Characterization of the precise mechanisms associated with homeostasis fueled by other types of stem cells has thus far lagged behind compared to research into the mechanisms of ISC-mediated homeostasis, and the importance of their regenerative capacity has only recently been appreciated. Here, we will describe several determinants that are involved in these stem cell self-renewal processes based on a series of recently published analyses.

First, we pay particular attention to the signaling involved in GsSC-mediated homeostasis. Xi R and colleagues found that Notch in GBs is responsible for CC or IC fate determination but not EE commitment and that damage-induced GsSC proliferation mediated by EGFR is associated with activation of VM-specific Vn and levels of the precursor-derived Spi [45]. Recently, Jasper H and colleagues focused on the Dpp-mediated acquisition of CCs. For example, the formation of CCs in the CCR requires Dpp, and defects in GsSC differentiation are associated with the loss of Dpp signaling pathway components, which does not occur in ISCs residing in the PM [141]. Furthermore, in another recent study, Jasper H and colleagues also found that suppression of Dad in the CCR triggered by ultrabithorax (Ubx) activation is responsible for Dpp expression, which is linked to CC acquisition [150]. Additionally, the phenotype of mis-differentiation epitomized by ectopic EC-like cells in the CCR, which are triggered by excessive JAK-STAT signaling, was well characterized in a recent study, suggesting an essential role for the JAK-STAT pathway in the proper differentiation of the CCR [151]. Altogether, these observations indicate that EGFR is closely associated with GsSC proliferation and that Notch, Dpp and the JAK-STAT pathway are the key factors regulating differentiation in the CCR. However, in-depth analyses are required to define additional regulators.

Second, our knowledge of GaSC and HSC-mediated regeneration is still rudimentary. According to Hou SX and colleagues, JAK-STAT and Hedgehog (Hh) are essential for GaSC proliferation and differentiation, respectively, and Wg affects GaSC regeneration [34]. However, the precise number of cell types present in the cardia remains unclear. Additionally, the rapidly proliferating progeny are generated via Wg-mediated slowly proliferating HSCs, and they differentiate into mature cells located in the posterior HPZ via Hh in response to environmental challenge [32]. These findings suggest that Hh is responsible for both GaSC and HSC differentiation. However, it remains largely unknown why the same signaling molecule would control different types of stem cell-mediated differentiation processes.

Third, besides GsSCs, GaSCs and HSCs, RNSCs have the unique capacity to maintain the self-renewal of MTs. RNSC fate is regulated by low Notch activation triggered by Delta-mediated *cis*-inhibition, resulting in STAT92E nuclear localization [152]. High Notch activation in renalblasts (RBs) controls the inhibition of STAT92E nuclear localization, causing RB differentiation [152]. Premature RNSC differentiation is induced by the loss of JAK-STAT, and excessive Upd levels contribute to increased RNSC division [36]. These results indicate that Notch is a key RB differentiation-promoting factor and that JAK-STAT regulates RNSC fate and proliferation but inhibits its differentiation. Cai Y and colleagues described the contribution of dMyc and CycE-mediated EGFR/MAPK, which functions independently of JAK-STAT, to RNSC proliferation; however, EGFR/MAPK is dispensable for RNSC fate determination [153]. Additionally, how normal stem cells are transformed into cancer stem cells was explained by Hou SX and colleagues, who proposed that loss of the tumor suppressors Salvador (Sav) or Scribble (Scrib) is responsible for tumorigenesis [154]. Thus, future work will provide rich mechanistic insights to enhance our understanding of the pathology of nephropathy.

To summarize, how these other stem cell types control tissue regeneration is difficult to comprehend, and the underlying research into these other stem cell-based inflammatory gastrointestinal diseases may ultimately be complex. A key question is whether there is a direct or indirect relationship with homeostasis mediated by these other stem cells? In this regard, we should pay more attention to the study of other types of stem cells that mediate intestinal homeostasis in the coming years.

## Conclusion and perspectives

In our review, we primarily discussed the “state of the art” findings of a large number of studies and provided a brief introduction to the global mechanistic framework underlying homeostasis in the adult *Drosophila* gastrointestinal tract. The purpose of this review was to identify several outstanding questions regarding stem cell biology mechanisms, the elucidation of which is of obvious value.

In-depth analyses are required to define the key determinants of tumor growth in specific regions, such as the gastric gland, in which it is unknown how stem cells provide a control point for epithelial renewal. Additionally, considering the high activity of stem cell-mediated intestinal responses, research investigating homeostasis has focused on female intestines. Little is known about the effects of stem cells on homeostasis in the adult male gastrointestinal tract. Whether the male intestines are armed with molecular mechanisms similar to those that have been found in the female intestines remains unclear. Indeed, studies focusing on the antiviral response have facilitated our understanding of the pathways that play pivotal roles in resistance to oral viral infection [155–157], such as RNA interference, the JAK-STAT pathway, and the NF-κB pathway. Exciting questions that have arisen from these preliminary studies include the following: how does viral infection manipulate stem cell activity, and what are the exact programs underlying virus-induced tissue pathology? Finally, several fundamental issues must still be addressed, such as “the molecular mechanism underlying the “cross-talk” between the adult gut and neighboring organs”, “how EEs are generated from ISCs” and “the beneficial and negative influences of the molecular dialogue underlying the host-microbiota interaction”. For example, the microbiota induces several homeostatic pathways in the gut, such as the Notch and JAK-STAT pathways [29]. Additionally, loss of the microbiota causes a decrease and an increase in the number of EBs and EEs, respectively, but this has no effect on the EC population [29]. In conclusion, research investigating stem cell behavior in the adult *Drosophila* gastrointestinal epithelium has experienced tremendous progress, which, from a clinical perspective, may allow us to elucidate the pathogenesis of different gastrointestinal and metabolic disorders in humans.

## Abbreviations

A1-P4: anterior1-posterior4
AM: anterior midgut
AMP: antimicrobial peptide
Apc: adenomatous polyposis coli
AS-C: acheate-scute complex
Ase: asense
ATM: ataxia telangiectasia-mutated
ATR: ATM- and RAD3-related
BMP: bone morphogenetic protein
CC: copper cell
CCR: copper cell region
Cdk4: cyclin-dependent kinase 4
Chn: Charlatan
Cic: Capicua
COPI: coat protein complex I
CycE: Cyclin E
Da: Daughterless
Dmrt: doublesex and mab-3 related transcription factor
Dpp: Decapentaplegic
DSS: dextran sulfate sodium
Dsx: doublesex
DUOX: dual oxidase
EB: enteroblast
EC: enterocyte
E-cad: E-cadherin
ECM: extracellular matrix
EE: enteroendocrine cell
EGFR: epidermal growth factor receptor
EMT: epithelial-mesenchymal transition
ER: endoplasmic reticulum
ERK: extracellular signal-regulated kinase
Esg: escargot
FCR: Fe cell region
Fru: fruitless
Fz: Frizzled
GaSC/GsSC: gastric stem cells
GB: gastroblast
Gce: Germ cell-expressed
Hh: Hedgehog
HPZ: hindgut proliferation zone
Hs3st-A: eparin sulfate 3-O sulfotransferase A
HSC: hindgut stem cell
IC: interstitial cell
Ilp3: insulin growth factor-like peptide 3
InR: insulin receptor
ISCs: intestinal stem cells
JAK-STAT: Janus kinase-signal transducer and activator of transcription
JH: juvenile hormone
JNK: Jun-N-terminal kinase
Keap1: Kelch-like ECH-associated protein 1
Krn: Keren
LFC: large flat cell
LFCR: large flat cell region
MAPK: mitogen-activated protein kinase
Met: Methoprene-tolerant
MM: middle midgut
MT: Malpighian tubule
PC: principal cell
Pcan: Perlecan
PERK: PKR-like ER kinase
PGN: peptidoglycan
Pink1: phosphatase and tensin homologue-induced putative kinase 1
PM: posterior midgut
Pnt: Pointed
PV: proventriculus
PVR: PDGF/VEGF receptor
R0-R5: region0-region5
RBs: renalblasts
RC: renalcyte
RNSCs: renal stem cells
ROS: rapid reactive oxide species
RyR: ryanodine receptor
Sav: Salvador
Sc: scute
SC: stellate cell
Scrib: Scribble
SOCS3: suppressor of cytokine signaling 3
STAT: signal transducer and activator of transcription
Stg: string
Sxl: Sex lethal
TA cell: transient amplifying cell
Tra: transformer
TRPA1: transient receptor potential A1
TSC: tuberous sclerosis complex
Ubx: ultrabithorax
Upd1: Unpaired 1
VM: visceral muscle
Vn: vein; Spi: Spitz
Wg: wingless
Wnt: Wingless and INT-1

## References

[1] Micchelli CA, Perrimon N (2006). Evidence that stem cells reside in the adult *Drosophila* midgut epithelium. Nature.

[2] Ohlstein B, Spradling A (2006). The adult *Drosophila* posterior midgut is maintained by pluripotent stem cells. Nature.

[3] Adams PD, Jasper H, Rudolph KL (2015). Aging-induced stem cell mutations as drivers for disease and cancer. Cell Stem Cell.

[4] Ulgherait M, Rana A, Rera M, Graniel J, Walker DW (2014). AMPK modulates tissue and organismal aging in a non-cell-autonomous manner. Cell Rep.

[5] Panayidou S, Apidianakis Y (2013). Regenerative inflammation: lessons from *Drosophila* intestinal epithelium in health and disease. Pathogens.

[6] Li Q, Ip YT (2015). More frequent than desired: Midgut stem cell somatic mutations. Cell Stem Cell.

[7] Shibata T, Maki K, Hadano J, Fujikawa T, Kitazaki K, Koshiba T, Kawabata S (2015). Crosslinking of a Peritrophic matrix protein protects gut epithelia from bacterial exotoxins. PLoS Pathog.

[8] Kuraishi T, Binggeli O, Opota O, Buchon N, Lemaitre B (2011). Genetic evidence for a protective role of the peritrophic matrix against intestinal bacterial infection in *Drosophila melanogaster*. Proc Natl Acad Sci U S A.

[9] You H, Lee WJ, Lee WJ (2014). Homeostasis between gut-associated microorganisms and the immune system in *Drosophila*. Curr Opin Immunol.

[10] Bosco-Drayon V, Poidevin M, Boneca IG, Narbonne-Reveau K, Royet J, Charroux B (2012). Peptidoglycan sensing by the receptor PGRP-LE in the *Drosophila* gut induces immune responses to infectious bacteria and tolerance to microbiota. Cell Host Microbe.

[11] Buchon N, Broderick NA, Poidevin M, Pradervand S, Lemaitre B (2009). *Drosophila* intestinal response to bacterial infection: activation of host defense and stem cell proliferation. Cell Host Microbe.

[12] Basset A, Khush RS, Braun A, Gardan L, Boccard F, Hoffmann JA (2000). The phytopathogenic bacteria Erwinia carotovora infects *Drosophila* and activates an immune response. Proc Natl Acad Sci U S A.

[13] Tzou P, Ohresser S, Ferrandon D, Capovilla M, Reichhart JM, Lemaitre B, Hoffmann JA, Imler JL (2000). Tissue-specific inducible expression of antimicrobial peptide genes in *Drosophila* surface epithelia. Immunity.

[14] Neyen C, Poidevin M, Roussel A, Lemaitre B (2012). Tissue- and ligand-specific sensing of gram-negative infection in *drosophila* by PGRP-LC isoforms and PGRP-LE. J Immunol.

[15] Jiang H, Patel PH, Kohlmaier A, Grenley MO, McEwen DG, Edgar BA (2009). Cytokine/Jak/Stat signaling mediates regeneration and homeostasis in the *Drosophila* midgut. Cell.

[16] Guo Z, Lucchetta E, Rafel N, Ohlstein B (2016). Maintenance of the adult *Drosophila* intestine: all roads lead to homeostasis. Curr Opin Genet Dev.

[17] Bonfini A, Liu X, Buchon N (2016). From pathogens to microbiota: how *Drosophila* intestinal stem cells react to gut microbes. Dev Comp Immunol.

[18] Dutta D, Dobson AJ, Houtz PL, Gläßer C, Revah J, Korzelius J, Patel PH, Edgar BA, Buchon N (2015). Regional cell-specific Transcriptome mapping reveals regulatory complexity in the adult *Drosophila* Midgut. Cell Rep.

[19] Casali A, Batlle E (2009). Intestinal stem cells in mammals and *Drosophila*. Cell Stem Cell.

[20] Lemaitre B, Miguel-Aliaga I (2013). The digestive tract of *Drosophila melanogaster*. Annu Rev Genet.

[21] Royet J (2011). Epithelial homeostasis and the underlying molecular mechanisms in the gut of the insect model *Drosophila melanogaster*. Cell Mol Life Sci.

[22] Shanbhag S, Tripathi S (2009). Epithelial ultrastructure and cellular mechanisms of acid and base transport in the *Drosophila* midgut. J Exp Biol.

[23] Affolter M, Barde Y (2007). Self-renewal in the fly kidney. Dev Cell.

[24] Singh SR, Hou SX (2008). Lessons learned about adult kidney stem cells from the malpighian tubules of *Drosophila*. J Am Soc Nephrol.

[25] Singh SR, Hou SX (2009). Multipotent stem cells in the Malpighian tubules of adult *Drosophila melanogaster*. J Exp Biol.

[26] Dow JA, Davies SA (2006). The Malpighian tubule: rapid insights from post-genomic biology. J Insect Physiol.

[27] Buchon N, Osman D, David FP, Fang HY, Boquete JP, Deplancke B, Lemaitre B (2013). Morphological and molecular characterization of adult midgut compartmentalization in *Drosophila*. Cell Rep.

[28] Marianes A, Spradling AC (2013). Physiological and stem cell compartmentalization within the *Drosophila* midgut. elife.

[29] Broderick NA, Buchon N, Lemaitre B (2014). Microbiota-induced changes in *drosophila melanogaster* host gene expression and gut morphology. MBio.

[30] O'Brien LE, Bilder D (2013). Beyond the niche: tissue-level coordination of stem cell dynamics. Annu Rev Cell Dev Biol.

[31] Zeng X, Chauhan C, Hou SX (2013). Stem cells in the *Drosophila* digestive system. Adv Exp Med Biol.

[32] Takashima S, Mkrtchyan M, Younossi-Hartenstein A, Merriam JR, Hartenstein V (2008). The behaviour of *Drosophila* adult hindgut stem cells is controlled by Wnt and Hh signalling. Nature.

[33] Fox DT, Spradling AC (2009). The *Drosophila* hindgut lacks constitutively active adult stem cells but proliferates in response to tissue damage. Cell Stem Cell.

[34] Singh SR, Zeng X, Zheng Z, Hou SX (2011). The adult *Drosophila* gastric and stomach organs are maintained by a multipotent stem cell pool at the foregut/midgut junction in the cardia (proventriculus). Cell Cycle.

[35] Strand M, Micchelli CA (2011). Quiescent gastric stem cells maintain the adult *Drosophila* stomach. Proc Natl Acad Sci U S A.

[36] Singh SR, Liu W, Hou SX (2007). The adult *Drosophila* malpighian tubules are maintained by multipotent stem cells. Cell Stem Cell.

[37] Biteau B, Hochmuth CE, Jasper H (2011). Maintaining tissue homeostasis: dynamic control of somatic stem cell activity. Cell Stem Cell.

[38] Guo Z, Ohlstein B. Stem cell regulation. Bidirectional Notch signaling regulates *Drosophila* intestinal stem cell multipotency. Science. 2015;350(6263):aab0988. 10.1126/science.aab0988 PMC5431284 26586765

[39] Zeng X, Hou SX (2015). Enteroendocrine cells are generated from stem cells through a distinct progenitor in the adult *Drosophila* posterior midgut. Development.

[40] Biteau B, Jasper H (2014). Slit/Robo signaling regulates cell fate decisions in the intestinal stem cell lineage of *Drosophila*. Cell Rep.

[41] Bardin AJ, Perdigoto CN, Southall TD, Brand AH, Schweisguth F (2010). Transcriptional control of stem cell maintenance in the *Drosophila* intestine. Development.

[42] Zeng X, Han L, Singh SR, Liu H, Neumüller RA, Yan D, Hu Y, Liu Y, Liu W, Lin X, Hou SX (2015). Genome-wide RNAi screen identifies networks involved in intestinal stem cell regulation in *Drosophila*. Cell Rep.

[43] Beehler-Evans R, Micchelli CA (2015). Generation of enteroendocrine cell diversity in midgut stem cell lineages. Development.

[44] Jiang H, Edgar BA (2011). Intestinal stem cells in the adult *Drosophila* midgut. Exp Cell Res.

[45] Wang C, Guo X, Xi R (2014). EGFR and notch signaling respectively regulate proliferative activity and multiple cell lineage differentiation of *Drosophila* gastric stem cells. Cell Res.

[46] Strand M, Micchelli CA (2013). Regional control of *Drosophila* gut stem cell proliferation: EGF establishes GSSC proliferative set point & controls emergence from quiescence. PLoS One.

[47] O'Brien LE (2013). Regional specificity in the *Drosophila* midgut: setting boundaries with stem cells. Cell Stem Cell.

[48] Lin G, Zhang X, Ren J, Pang Z, Wang C, Xu N, Xi R (2013). Integrin signaling is required for maintenance and proliferation of intestinal stem cells in *Drosophila*. Dev Biol.

[49] Okumura T, Takeda K, Taniguchi K, Adachi-Yamada T (2014). βν integrin inhibits chronic and high level activation of JNK to repress senescence phenotypes in *Drosophila* adult midgut. PLoS One.

[50] Kapuria S, Karpac J, Biteau B, Hwangbo D, Jasper H (2012). Notch-mediated suppression of TSC2 expression regulates cell differentiation in the *Drosophila* intestinal stem cell lineage. PLoS Genet.

[51] Okumura T, Takeda K, Kuchiki M, Akaishi M, Taniguchi K, Adachi-Yamada T (2016). GATAe regulates intestinal stem cell maintenance and differentiation in *Drosophila* adult midgut. Dev Biol.

[52] Amcheslavsky A, Nie Y, Li Q, He F, Tsuda L, Markstein M, Ip YT (2014). Gene expression profiling identifies the zinc-finger protein charlatan as a regulator of intestinal stem cells in *Drosophila*. Development.

[53] Tian A, Jiang J (2014). Intestinal epithelium-derived BMP controls stem cell self-renewal in *Drosophila* adult midgut. elife.

[54] Korzelius J, Naumann SK, Loza-Coll MA, Chan JS, Dutta D, Oberheim J, Gläßer C, Southall TD, Brand AH, Jones DL, Edgar BA (2014). Escargot maintains stemness and suppresses differentiation in *Drosophila* intestinal stem cells. EMBO J.

[55] Loza-Coll MA, Southall TD, Sandall SL, Brand AH, Jones DL (2014). Regulation of *Drosophila* intestinal stem cell maintenance and differentiation by the transcription factor escargot. EMBO J.

[56] Antonello ZA, Reiff T, Ballesta-Illan E, Dominguez M (2015). Robust intestinal homeostasis relies on cellular plasticity in enteroblasts mediated by miR-8-escargot switch. EMBO J.

[57] Osman D, Buchon N, Chakrabarti S, Huang YT, Su WC, Poidevin M, Tsai YC, Lemaitre B (2012). Autocrine and paracrine unpaired signaling regulate intestinal stem cell maintenance and division. J Cell Sci.

[58] Biteau B, Hochmuth CE, Jasper H (2008). JNK activity in somatic stem cells causes loss of tissue homeostasis in the aging *Drosophila* gut. Cell Stem Cell.

[59] Lin G, Xu N, Xi R (2008). Paracrine wingless signalling controls self-renewal of *Drosophila* intestinal stem cells. Nature.

[60] Goulas S, Conder R, Knoblich JA (2012). The par complex and integrins direct asymmetric cell division in adult intestinal stem cells. Cell Stem Cell.

[61] Li Z, Zhang Y, Han L, Shi L, Lin X (2013). Trachea-derived dpp controls adult midgut homeostasis in *Drosophila*. Dev Cell.

[62] Guo Z, Driver I, Ohlstein B (2013). Injury-induced BMP signaling negatively regulates *Drosophila* midgut homeostasis. J Cell Biol.

[63] Quan Z, Sun P, Lin G, Xi R (2013). TSC1/2 regulates intestinal stem cell maintenance and lineage differentiation through Rheb-TORC1-S6K but independently of nutritional status or notch regulation. J Cell Sci.

[64] Jin Y, Patel PH, Kohlmaier A, Pavlovic B, Zhang C, Edgar BA (2017). Intestinal stem cell pool regulation in *Drosophila*. Stem Cell Reports..

[65] Hudry B, Khadayate S, Miguel-Aliaga I (2016). The sexual identity of adult intestinal stem cells controls organ size and plasticity. Nature.

[66] Robinett CC, Vaughan AG, Knapp JM, Baker BS (2010). Sex and the single cell. II. There is a time and place for sex. PLoS Biol.

[67] Clough E, Jimenez E, Kim YA, Whitworth C, Neville MC, Hempel LU, Pavlou HJ, Chen ZX, Sturgill D, Dale RK, Smith HE, Przytycka TM, Goodwin SF, Van Doren M, Oliver B (2014). Sex- and tissue-specific functions of *Drosophila* doublesex transcription factor target genes. Dev Cell.

[68] Regan JC, Khericha M, Dobson AJ, Bolukbasi E, Rattanavirotkul N, Partridge L (2016). Sex difference in pathology of the ageing gut mediates the greater response of female lifespan to dietary restriction. elife.

[69] Chen CH, Luhur A, Sokol N (2015). Lin-28 promotes symmetric stem cell division and drives adaptive growth in the adult *Drosophila* intestine. Development.

[70] Montagne C, Gonzalez-Gaitan M (2014). Sara endosomes and the asymmetric division of intestinal stem cells. Development.

[71] Yamashita YM, Fuller MT (2008). Asymmetric centrosome behavior and the mechanisms of stem cell division. J Cell Biol.

[72] Yamashita Y (2009). Asymmetric stem cell division and pathology: insights from *Drosophila* stem cell systems. J Pathol.

[73] Januschke J, Gonzalez C (2008). *Drosophila* asymmetric division, polarity and cancer. Oncogene.

[74] Knoblich JA (2008). Mechanisms of asymmetric stem cell division. Cell.

[75] Hou SX (2010). Intestinal stem cell asymmetric division in the *Drosophila* posterior midgut. J Cell Physiol.

[76] O'Brien LE, Soliman SS, Li X, Bilder D (2011). Altered modes of stem cell division drive adaptive intestinal growth. Cell.

[77] Takashima S, Gold D, Hartenstein V (2013). Stem cells and lineages of the intestine: a developmental and evolutionary perspective. Dev Genes Evol.

[78] Wilson AA, Kotton DN (2008). Another notch in stem cell biology: *Drosophila* intestinal stem cells and the specification of cell fates. BioEssays.

[79] Choi NH, Kim JG, Yang DJ, Kim YS, Yoo MA (2008). Age-related changes in *Drosophila* midgut are associated with PVF2, a PDGF/VEGF-like growth factor. Aging Cell.

[80] Park JS, Na HJ, Pyo JH, Jeon HJ, Kim YS, Yoo MA (2015). Requirement of ATR for maintenance of intestinal stem cells in aging *Drosophila*. Aging (Albany NY).

[81] Lucchetta EM, Ohlstein B (2012). The *Drosophila* midgut: a model for stem cell driven tissue regeneration. Wiley Interdiscip Rev Dev Biol.

[82] Patel PH, Dutta D, Edgar BA (2015). Niche appropriation by *Drosophila* intestinal stem cell tumours. Nat Cell Biol.

[83] Biteau B, Jasper H (2011). EGF signaling regulates the proliferation of intestinal stem cells in *Drosophila*. Development.

[84] Buchon N, Broderick NA, Kuraishi T, Lemaitre B (2010). *Drosophila* EGFR pathway coordinates stem cell proliferation and gut remodeling following infection. BMC Biol.

[85] Jiang H, Grenley MO, Bravo MJ, Blumhagen RZ, Edgar BA (2011). EGFR/Ras/MAPK signaling mediates adult midgut epithelial homeostasis and regeneration in *Drosophila*. Cell Stem Cell.

[86] Scopelliti A, Cordero JB, Diao F, Strathdee K, White BH, Sansom OJ, Vidal M (2014). Local control of intestinal stem cell homeostasis by enteroendocrine cells in the adult *Drosophila* midgut. Curr Biol.

[87] Jin Y, Ha N, Forés M, Xiang J, Gläßer C, Maldera J, Jiménez G, Edgar BA (2015). EGFR/Ras signaling controls *Drosophila* intestinal stem cell proliferation via Capicua-regulated genes. PLoS Genet.

[88] Buchon N, Broderick NA, Chakrabarti S, Lemaitre B (2009). Invasive and indigenous microbiota impact intestinal stem cell activity through multiple pathways in *Drosophila*. Genes Dev.

[89] Zhou F, Rasmussen A, Lee S, Agaisse H (2013). The UPD3 cytokine couples environmental challenge and intestinal stem cell division through modulation of JAK/STAT signaling in the stem cell microenvironment. Dev Biol.

[90] Liu W, Singh SR, Hou SX (2010). JAK-STAT is restrained by notch to control cell proliferation of the *Drosophila* intestinal stem cells. J Cell Biochem.

[91] Lin G, Xu N, Xi R (2010). Paracrine unpaired signaling through the JAK/STAT pathway controls self-renewal and lineage differentiation of *drosophila* intestinal stem cells. J Mol Cell Biol.

[92] Xu N, Wang SQ, Tan D, Gao Y, Lin G, Xi R (2011). EGFR, wingless and JAK/STAT signaling cooperatively maintain *Drosophila* intestinal stem cells. Dev Biol.

[93] Chakrabarti S, Dudzic JP, Li X, Collas EJ, Boquete JP, Lemaitre B (2016). Remote control of intestinal stem cell activity by Haemocytes in *Drosophila*. PLoS Genet.

[94] Choi NH, Lucchetta E, Ohlstein B (2011). Nonautonomous regulation of *Drosophila* midgut stem cell proliferation by the insulin-signaling pathway. Proc Natl Acad Sci U S A.

[95] Amcheslavsky A, Song W, Li Q, Nie Y, Bragatto I, Ferrandon D, Perrimon N, Ip YT (2014). Enteroendocrine cells support intestinal stem-cell-mediated homeostasis in *Drosophila*. Cell Rep.

[96] Lin G, Xi R (2008). Intestinal stem cell, muscular niche and wingless signaling. Fly (Austin).

[97] Belenkaya TY, Wu Y, Tang X, Zhou B, Cheng L, Sharma YV, Yan D, Selva EM, Lin X (2008). The retromer complex influences Wnt secretion by recycling wntless from endosomes to the trans-Golgi network. Dev Cell.

[98] Wang C, Zhao R, Huang P, Yang F, Quan Z, Xu N, Xi R (2013). APC loss-induced intestinal tumorigenesis in *Drosophila*: roles of Ras in Wnt signaling activation and tumor progression. Dev Biol.

[99] Lee WC, Beebe K, Sudmeier L, Micchelli CA (2009). Adenomatous polyposis coli regulates *Drosophila* intestinal stem cell proliferation. Development.

[100] Cordero JB, Stefanatos RK, Scopelliti A, Vidal M, Sansom OJ (2012). Inducible progenitor-derived wingless regulates adult midgut regeneration in *Drosophila*. EMBO J.

[101] Tan S, Barker N (2015). Epithelial stem cells and intestinal cancer. Semin Cancer Biol.

[102] Korinek V, Barker N, Moerer P, van Donselaar E, Huls G, Peters PJ, Clevers H (1998). Depletion of epithelial stem-cell compartments in the small intestine of mice lacking Tcf-4. Nat Genet.

[103] Clevers H (2006). Wnt/beta-catenin signaling in development and disease. Cell.

[104] Sato T, van Es JH, Snippert HJ, Stange DE, Vries RG, van den Born M, Barker N, Shroyer NF, van de Wetering M, Clevers H (2011). Paneth cells constitute the niche for Lgr5 stem cells in intestinal crypts. Nature.

[105] Karin M (2008). The IkappaB kinase-a bridge between inflammation and cancer. Cell Res.

[106] Rigby RJ, Simmons JG, Greenhalgh CJ, Alexander WS, Lund PK (2007). Suppressor of cytokine signaling 3 (SOCS3) limits damage-induced crypt hyper-proliferation and inflammation-associated tumorigenesis in the colon. Oncogene.

[107] Hochmuth CE, Biteau B, Bohmann D, Jasper H (2011). Redox regulation by Keap1 and Nrf2 controls intestinal stem cell proliferation in *Drosophila*. Cell Stem Cell.

[108] Xu C, Luo J, He L, Montell C, Perrimon N. Oxidative stress induces stem cell proliferation via TRPA1/RyR-mediated Ca^2+^ signaling in the *Drosophila* midgut. Elife. 2017;6:e22441. 10.7554/eLife.22441 PMC5451214 28561738

[109] Pan D (2010). The hippo signaling pathway in development and cancer. Dev Cell.

[110] Ren F, Wang B, Yue T, Yun EY, Ip YT, Jiang J (2010). Hippo signaling regulates *Drosophila* intestine stem cell proliferation through multiple pathways. Proc Natl Acad Sci U S A.

[111] Shaw RL, Kohlmaier A, Polesello C, Veelken C, Edgar BA, Tapon N (2010). The hippo pathway regulates intestinal stem cell proliferation during *Drosophila* adult midgut regeneration. Development.

[112] Staley BK, Irvine KD (2010). Warts and Yorkie mediate intestinal regeneration by influencing stem cell proliferation. Curr Biol.

[113] Staley BK, Irvine KD (2012). Hippo signaling in *Drosophila*: recent advances and insights. Dev Dyn.

[114] Karpowicz P, Perez J, Perrimon N (2010). The hippo tumor suppressor pathway regulates intestinal stem cell regeneration. Development.

[115] Bond D, Foley E (2012). Autocrine platelet-derived growth factor-vascular endothelial growth factor receptor-related (Pvr) pathway activity controls intestinal stem cell proliferation in the adult *Drosophila* midgut. J Biol Chem.

[116] Deng H, Gerencser AA, Jasper H (2015). Signal integration by ca(2+) regulates intestinal stem-cell activity. Nature.

[117] Wang L, Ryoo HD, Qi Y, Jasper H (2015). PERK limits *Drosophila* lifespan by promoting intestinal stem cell proliferation in response to ER stress. PLoS Genet.

[118] Koehler CL, Perkins GA, Ellisman MH, Jones DL. Pink1 and Parkin regulate *Drosophila* intestinal stem cell proliferation during stress and aging. J Cell Biol. 2017;216(8):2315–27. 10.1083/jcb.201610036 PMC5551703 28663346

[119] Rera M, Clark RI, Walker DW (2012). Intestinal barrier dysfunction links metabolic and inflammatory markers of aging to death in *Drosophila*. Proc Natl Acad Sci U S A.

[120] Reiff T, Jacobson J, Cognigni P, Antonello Z, Ballesta E, Tan KJ, Yew JY, Dominguez M, Miguel-Aliaga I (2015). Endocrine remodelling of the adult intestine sustains reproduction in *Drosophila*. elife.

[121] You J, Zhang Y, Li Z, Lou Z, Jin L, Lin X (2014). *Drosophila* perlecan regulates intestinal stem cell activity via cell-matrix attachment. Stem Cell Reports.

[122] Guo Y, Li Z, Lin X (2014). Hs3st-a and Hs3st-B regulate intestinal homeostasis in *Drosophila* adult midgut. Cell Signal.

[123] Huang X, Shi L, Cao J, He F, Li R, Zhang Y, Miao S, Jin L, Qu J, Li Z, Lin X (2014). The sterile 20-like kinase tao controls tissue homeostasis by regulating the hippo pathway in *Drosophila* adult midgut. J Genet Genomics..

[124] Meng FW, Biteau B (2015). A sox transcription factor is a critical regulator of adult stem cell proliferation in the *Drosophila* intestine. Cell Rep.

[125] Zhai Z, Kondo S, Ha N, Boquete JP, Brunner M, Ueda R, Lemaitre B (2015). Accumulation of differentiating intestinal stem cell progenies drives tumorigenesis. Nat Commun.

[126] Huang H, Li J, Hu L, Ge L, Ji H, Zhao Y, Zhang L (2014). Bantam is essential for *Drosophila* intestinal stem cell proliferation in response to Hippo signaling. Dev Biol.

[127] Brunton VG, Ozanne BW, Paraskeva C, Frame MC (1997). A role for epidermal growth factor receptor, c-Src and focal adhesion kinase in an in vitro model for the progression of colon cancer. Oncogene.

[128] Kohlmaier A, Fassnacht C, Jin Y, Reuter H, Begum J, Dutta D, Edgar BA (2015). Src kinase function controls progenitor cell pools during regeneration and tumor onset in the *Drosophila* intestine. Oncogene.

[129] Li Q, Li S, Mana-Capelli S, Roth Flach RJ, Danai LV, Amcheslavsky A, Nie Y, Kaneko S, Yao X, Chen X, Cotton JL, Mao J, McCollum D, Jiang J, Czech MP, Xu L, Ip YT (2014). The conserved misshapen-warts-Yorkie pathway acts in enteroblasts to regulate intestinal stem cells in *Drosophila*. Dev Cell.

[130] Yedvobnick B, Moberg K (2010). Linking model systems to cancer therapeutics: the case of mastermind. Dis Model Mech.

[131] Markstein M (2013). Modeling colorectal cancer as a 3-dimensional disease in a dish: the case for drug screening using organoids, zebrafish, and fruit flies. Drug Discov Today Technol.

[132] Markstein M, Dettorre S, Cho J, Neumüller RA, Craig-Müller S, Perrimon N (2014). Systematic screen of chemotherapeutics in *Drosophila* stem cell tumors. Proc Natl Acad Sci U S A.

[133] Singh SR, Zeng X, Zhao J, Liu Y, Hou G, Liu H, Hou SX (2016). The lipolysis pathway sustains normal and transformed stem cells in adult *Drosophila*. Nature.

[134] Kim SH, Lee WJ (2014). Role of DUOX in gut inflammation: lessons from *Drosophila* model of gut-microbiota interactions. Front Cell Infect Microbiol.

[135] Amcheslavsky A, Jiang J, Ip YT (2009). Tissue damage-induced intestinal stem cell division in *Drosophila*. Cell Stem Cell.

[136] Takeishi A, Kuranaga E, Tonoki A, Misaki K, Yonemura S, Kanuka H, Miura M (2013). Homeostatic epithelial renewal in the gut is required for dampening a fatal systemic wound response in *Drosophila*. Cell Rep.

[137] Capo F, Charroux B, Royet J (2016). Bacteria sensing mechanisms in *Drosophila* gut: local and systemic consequences. Dev Comp Immunol.

[138] Perdigoto CN, Bardin AJ (2013). Sending the right signal: notch and stem cells. Biochim Biophys Acta.

[139] Andriatsilavo M, Gervais L, Fons C, Bardin AJ (2013). The *Drosophila* midgut as a model to study adult stem cells. Med Sci (Paris).

[140] Zhou J, Florescu S, Boettcher AL, Luo L, Dutta D, Kerr G, Cai Y, Edgar BA, Boutros M (2015). Dpp/Gbb signaling is required for normal intestinal regeneration during infection. Dev Biol.

[141] Li H, Qi Y, Jasper H (2013). Dpp signaling determines regional stem cell identity in the regenerating adult *Drosophila* gastrointestinal tract. Cell Rep.

[142] Park JS, Kim YS, Yoo MA (2009). The role of p38b MAPK in age-related modulation of intestinal stem cell proliferation and differentiation in *Drosophila*. Aging (Albany NY).

[143] Xiang J, Bandura J, Zhang P, Jin Y, Reuter H, Edgar BA (2017). EGFR-dependent TOR-independent endocycles support *Drosophila* gut epithelial regeneration. Nat Commun.

[144] Chen J, Xu N, Huang H, Cai T, Xi R. A feedback amplification loop between stem cells and their progeny promotes tissue regeneration and tumorigenesis. Elife. 2016;5:e14330. 10.7554/eLife.14330 PMC4905741 27187149

[145] Zhai Z, Boquete JP, Lemaitre B (2017). A genetic framework controlling the differentiation of intestinal stem cells during regeneration in *Drosophila*. PLoS Genet.

[146] Zeng X, Lin X, Hou SX (2013). The Osa-Containing SWI/SNF chromatin-remodeling complex regulates stem cell commitment in the adult *Drosophila* intestine. Development.

[147] Wang C, Guo X, Dou K (2015). Chen H1, xi R. Ttk69 acts as a master repressor of enteroendocrine cell specification in *Drosophila* intestinal stem cell lineages. Development.

[148] Sallé J, Gervais L, Boumard B, Stefanutti M, Siudeja K, Bardin AJ. Intrinsic regulation of enteroendocrine fate by Numb. EMBO J. 2017;36(13):1928–45. 10.15252/embj.201695622 PMC5494454 28533229

[149] Adlesic M, Frei C, Frew IJ (2016). Cdk4 functions in multiple cell types to control *Drosophila* intestinal stem cell proliferation and differentiation. Biol Open.

[150] Li H, Qi Y, Jasper H. Ubx dynamically regulates Dpp signaling by repressing Dad expression during copper cell regeneration in the adult *Drosophila* midgut. Dev Biol. 2016;419(2):373–81. 10.1016/j.ydbio.2016.08.027 PMC5681348 27570230

[151] Li H, Qi Y, Jasper H (2016). Preventing age-related decline of gut compartmentalization limits microbiota Dysbiosis and extends lifespan. Cell Host Microbe.

[152] Li Z, Liu S, Cai Y (2014). Differential notch activity is required for homeostasis of malpighian tubules in adult *Drosophila*. J Genet Genomics..

[153] Li Z, Liu S, Cai Y (2015). EGFR/MAPK signaling regulates the proliferation of *Drosophila* renal and nephric stem cells. J Genet Genomics.

[154] Zeng X, Singh SR, Hou D, Hou SX (2010). Tumor suppressors Sav/Scrib and oncogene Ras regulate stem-cell transformation in adult *Drosophila* malpighian tubules. J Cell Physiol.

[155] Sabin LR, Hanna SL, Cherry S (2010). Innate antiviral immunity in *Drosophila*. Curr Opin Immunol.

[156] Merkling SH, van Rij RP (2013). Beyond RNAi: antiviral defense strategies in *Drosophila* and mosquito. J Insect Physiol.

[157] Ferreira ÁG, Naylor H, Esteves SS, Pais IS, Martins NE, Teixeira L (2014). The toll-dorsal pathway is required for resistance to viral oral infection in *Drosophila*. PLoS Pathog.

